# Long-acting anti-inflammatory injectable DEX-Gel with sustained release and self-healing properties regulates T_H_1/T_H_2 immune balance for minimally invasive treatment of allergic rhinitis

**DOI:** 10.1186/s12951-024-02306-w

**Published:** 2024-02-06

**Authors:** Li Dai, Bin Liu, Jiangtao Lin, Yongquan Jiang, Yuanyuan Li, Zhuowei Yao, Silin Shen, Yiming Jiang, Yourong Duan, Jiping Li

**Affiliations:** 1 State Key Laboratory of Systems Medicine for Cancer, Shanghai Cancer Institute, Ren Ji Hospital, Shanghai Jiao Tong University School of Medicine, Shanghai, 200032 China; 2 Department of Otolaryngology, Ren Ji Hospital, Shanghai Jiao Tong University School of Medicine, Shanghai, 200127 China

**Keywords:** Allergic rhinitis, Sustained-release anti-inflammatory, T_H_1/T_H_2 immune balance, Injectable gel, Minimally invasive treatment

## Abstract

**Background:**

Allergic rhinitis (AR) is a prevalent immune-related allergic disease, and corticosteroid nasal sprays serve as the primary treatment for this patient population. However, their short duration of efficacy and frequent administration pose challenges, leading to drug wastage and potential adverse effects. To overcome these limitations, we devised a novel approach to formulate DEX-Gel by incorporating dexamethasone (DEX) into a blend of Pluronic F127, stearic acid (SA), and polyethylene glycol 400 (PEG400) to achieve sustained-release treatment for AR.

**Results:**

Following endoscopic injection into the nasal mucosa of AR rats, DEX-Gel exhibited sustained release over a 14-day period. In vivo trials employing various assays, such as flow cytometry (FC), demonstrated that DEX-Gel not only effectively managed allergic symptoms but also significantly downregulated helper T-cells (T_H_) 2 and T_H_2-type inflammatory cytokines (e.g., interleukins 4, 5, and 13). Additionally, the T_H_1/T_H_2 cell ratio was increased.

**Conclusion:**

This innovative long-acting anti-inflammatory sustained-release therapy addresses the T_H_1/T_H_2 immune imbalance, offering a promising and valuable approach for the treatment of AR and other inflammatory nasal diseases.

**Graphical Abstract:**

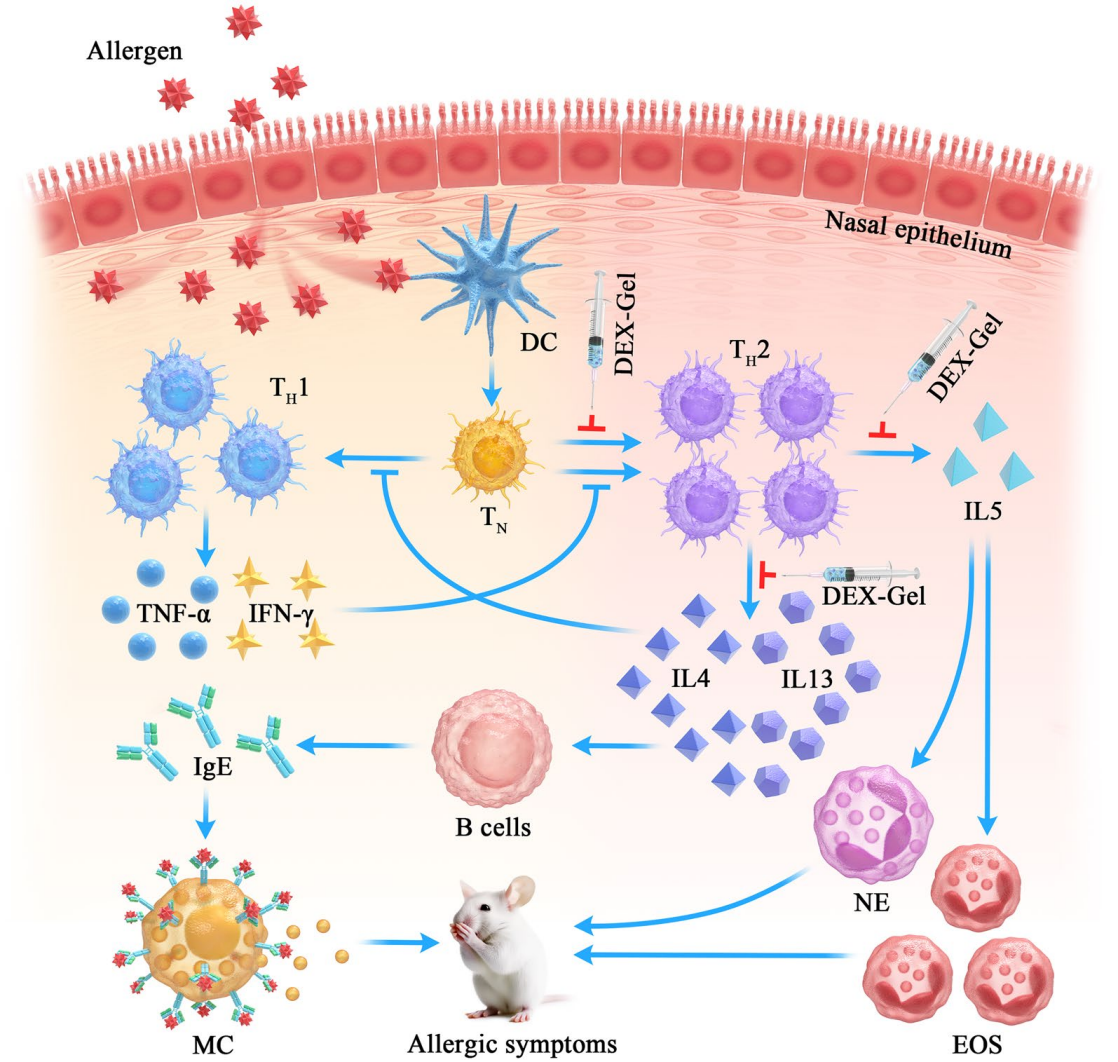

**Supplementary Information:**

The online version contains supplementary material available at 10.1186/s12951-024-02306-w.

## Introduction

Allergic rhinitis (AR) stands as one of the most prevalent global diseases, affecting 10–40% of the population [1]. Characterized by allergic reactions in the nasal mucosa, key symptoms encompass itching, nasal congestion, runny nose, and sneezing [2, 3]. In China, the standardized prevalence of adult AR ranges from 8 to 21.4% [4]. Moreover, in the northern grassland region, where seasonal pollen concentrations soar, the self-reported prevalence of pollen-induced AR (PiAR) reaches as high as 32.4% [5]. AR exerts substantial adverse effects on social life, academic performance, and work productivity, imposing a considerable economic burden on society [6–8].

It is now understood that AR, an immune-related disease, involves diverse mechanisms [9]. Helper T cells (T_H_) 1 and 2, two distinct types of T_H_ cells, contribute to an imbalanced T_H_1 and T_H_2 immunity, recognized as a pivotal pathological mechanism of AR [10, 11]. In type I hypersensitivity, the T_H_2 immune response releases cytokines such as interleukin-4 (IL4), IL5, and IL13. These cytokines overstimulate immunoglobulin E (IgE), triggering eosinophil infiltration and heightened mucus secretion, ultimately manifesting various AR symptoms [12, 13].

Commonly employed treatments for AR include nasal spray/oral glucocorticoids, antihistamines, leukotriene receptor antagonists, and allergen-specific immunotherapy (AIT). Among these, glucocorticoids, especially dexamethasone (DEX), have emerged as the most effective in symptom control. Despite nasal glucocorticoids mitigating systemic adverse effects associated with oral intake, their effectiveness is limited due to the brief contact time on the nasal mucosa, influenced by nasal cilia and mucus. This often leads to incomplete drug absorption before clearance [14, 15]. Improper administration and frequent nasal spraying not only impact medication dosage but also induce side effects such as nasal irritation, stinging, and rhinorrhea [16]. Consequently, there is a compelling need for nasal formulations demonstrating optimal clinical efficacy, prolonged anti-inflammatory effects, and excellent safety.

**Scheme 1:**
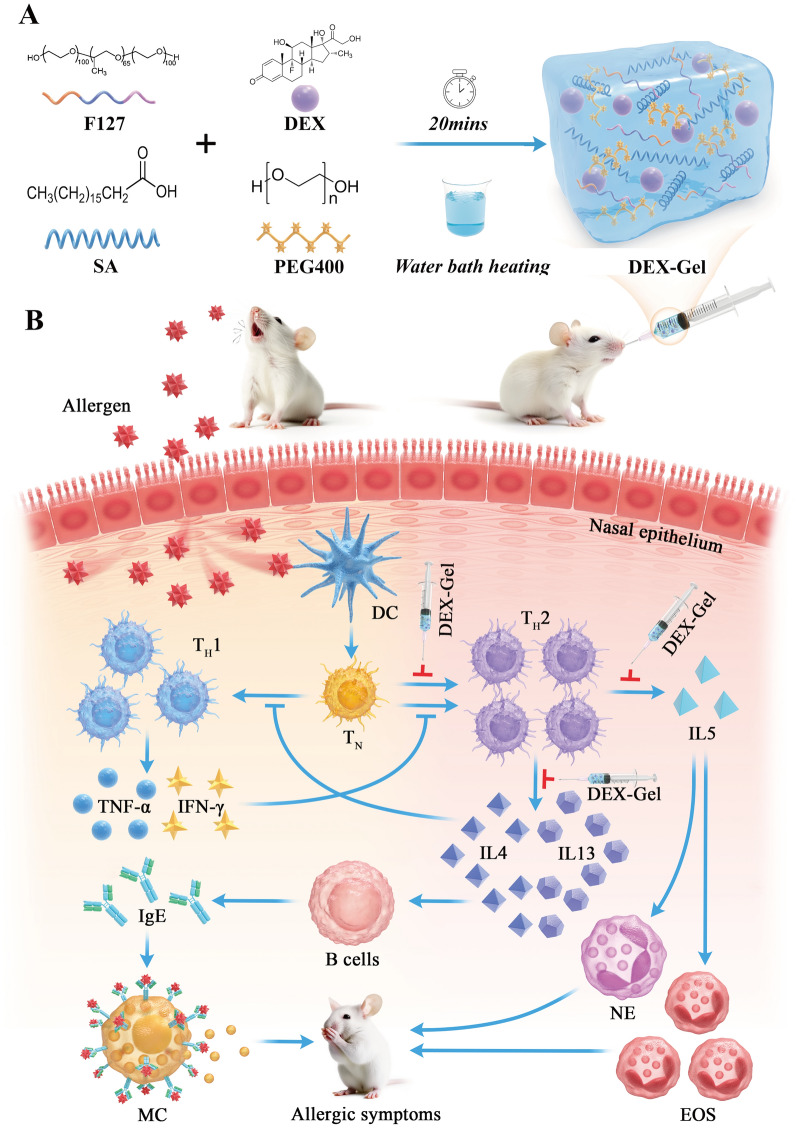
Schematic illustration for **A** DEX-Gel synthesis and **B** its mechanism of regulating T_H_1/T_H_2 immune balance in allergic rhinitis. *DC* dendritic cells, *T*_*N*_ naive T cells, *TH* helper T cells, *MC* mast cells, *NE* neutrophils, *EOS* eosinophils, *IgE* immunoglobulin E

In recent years, the application of biomedical materials has made rapid progress [17–20]. While some bioadhesive formulations encapsulating DEX have been developed to extend drug residence time in the nasal mucosa, the duration remains unsatisfactory, lasting only a few hours or days [21–23]. Addressing this limitation, we designed a gel (referred to as Gel) utilizing Pluronic F127, stearic acid (SA), and polyethylene glycol 400 (PEG400), and the DEX-encapsulated Gel is referred to as DEX-Gel. Pluronic F127 and SA form the main backbone structure of the gel, PEG400 serves as a common solvent and increases the solubility of DEX. DEX-Gel spontaneously restores its three-dimensional structure at room temperature, providing a sustained release for up to 14 days. The protracted and gradual release of submucosal DEX-Gel not only mitigates systemic side effects and the need for repeated administration but also modulates the AR pathogenic microenvironment. This, in turn, addresses the T_H_1/T_H_2 imbalance, presenting a valuable and promising avenue for AR treatments (Scheme 1).

## Materials and methods

### Preparation and morphological characterization of DEX-Gel

DEX powder, Pluronic F127, SA, and PEG400 were purchased from Shanghai Macklin Biochemical Co. DEX, Pluronic F127, SA were dissolved in 1 mL volume of PEG400 by heating in a water bath to obtain the DEX-Gel. The lyophilized DEX-Gel was fixed on a copper table with conductive adhesive and then sprayed with a thin layer of gold. The physical characteristics of the gel were observed under a scanning electron microscope (SEM) (HITACHI).

### Injectability and rheological test of the DEX-Gel

The injectability of the gel was confirmed by extruding it through a Φ 1 mm needle. The rheological test of the gel was performed as follows. First, the critical strain value required to break the gel network and change it to the solution state was determined by continuously varying the stress (0.1–100%) and frequency (0.1–100 HZ). Next, to verify the self-healing properties of the gels, dynamic stress-viscosity tests were performed on the gels at a frequency of 1 rad s^−1^. A low stress (γ = 1%) was applied to the gels for the first 600 s, followed by the application of a high stress (γ = 100%) for 120 s. The test was repeated for 3 cycles. Finally, the viscosity changes of both gels were examined by different shear stress (0.1%-100%) and shear rates (0.1–100 s^−1^).

### In vitro gel degradation and drug release assay and in vivo gel degradation assay

After making the gel, the weight of the initial gel sample was recorded as Wa. Then, an equal volume of deionized water was added, and the gel was incubated in a thermostatic shaker at 120 r min^−1^ at 37 °C. The deionized water was replaced at different time points, and the weight of the gel, Wb, was recorded. The percentage of residual mass was calculated according to the following metric: percentage of residual mass = Wb/Wa × 100%.

To test the release of DEX from the DEX-Gel, 200 µL DEX-Gel was first soaked in 10 mL of phosphate-buffered saline (PBS) solution with pH = 7.4 and incubated in a constant temperature shaker at 120r min-1, 37 °C. At different time points, we transferred 50 µL of the release solution and added 50 µL of fresh PBS. the concentration of samples released from the DEX-Gel was detected by HPLC (Agilent 1200, Germany), and a concentration curve was plotted using the standard DEX (Additional file 1: Fig. S1).

To further validate the in vivo degradation of the gel, we injected 200 µL DEX-Gel subcutaneously in rats and detected the gel degradation at different time points.

### CCK8 cytotoxicity assay

The effects of DEX-Gel on cell viability were assessed by a CCK-8 assay (Dojindo, Japan). L929 (10^4^ cells/well) were seeded in a 96-well plate and cultured for 12 h. Further, the cells were treated with various concentrations of DEX-Gel extraction at different times. 100 µl of 10% CCK-8 solution was added to each well, then the cells were incubated for 2 h. The absorbance was measured at 450 nm with an enzyme labeling instrument (Synergy H4, USA). Cell viability was normalized to the control group.

### Hemolysis test

Blood was incubated with distilled water (positive tubes), PBS (negative tubes), and different concentrations of DEX-Gel extraction at 37 °C for 60 min, respectively. After centrifugation, the absorbance of the supernatant in each group at 540 nm was determined. The hemolysis rate (%) was calculated by the following equation:

$${\text{Hemolysis}}\,\left( \% \right)\, = \,\left[ {\left( {{\text{ODx}}\, - \,{\text{ODo}}} \right)\,/\,\left( {{\text{ODy}}\, - \,{\text{ODo}}} \right)} \right]\times 100$$

where OD_x_, ODo, and ODy are the absorbance values of the DEX-Gel, diluted blood in negative tubes, and diluted blood in positive tubes, respectively. The OD values of the negative tubes were < 0.03, and the OD values of the positive tubes were between 0.6 and 1.1.

### Foreign body reaction and ciliotoxicity test of DEX-Gel

Rats were injected with 200 μL DEX-gel under the nasal mucosa. On days 0, 1, 7, and 14, the mucosa was stripped for HE staining to count neutrophils, and the mucosal IL4 levels were measured by flow cytometry.

12 *Bufo chinensis* were purchased from Jiaxiang Huarong Breeding Specialized Cooperative (Shandong, China), Production License: 93370829MA3N4L6N9B. After the spinal cord of the toad was crushed, the mucous membrane of the palate of about 3 mm × 3 mm was removed by surgical clipping. 200 μL PBS or different concentrations of DEX-Gel extract was added to the surface of the mucosa, and observe the movement of the mucosal cilia was observed under a 40 × light microscope. The slides were removed at appropriate intervals until the cilia stopped moving, and the duration of cilia movement was recorded.

### AR rat model establishment

42 SD female rats (6–8 weeks) were purchased from the Animal Experiment Center of Ren Ji Hospital (Ren Ji Hospital, Shanghai, China). This study was approved by the Ethics Committee of Ren Ji Hospital (approval number: RJ2023- 134A). Thirty SD rats were randomly divided into groups Con, AR, Gel, Rhinocort, DEX and DEX-Gel (n = 5 in each group). Another 12 rats were used for DEX-Gel in vivo degradation assay and foreign body reaction assay. All thirty SD rats were sensitized with 50 µg ovalbumin (OVA) together with 4 mg Alum (400 µL) by intraperitoneal injection on days 0, 2, 4, 6, 8, 10, 12, and 14. On days 21–28, 25 rats (except group Con) were challenged by stimulation with 1250 µg/20 µL OVA daily by intranasal administration. On day 28, rats in the group AR had an allergy score of > 5 and the AR rat model was successfully established.

### Topical administration treatment

From day 21 to 28, Groups Rhinocort or DEX were treated with nasal drops of 50 µg/20 µL Rhinocort or DEX, group AR were challenged with PBS. Groups Gel or DEX-Gel were treated with nasal submucosal injection of 800 µg/200 µL Gel or DEX-Gel on day 21. SD rats were anesthetized by inhalation of isoflurane (5%, 0.3 L/min), and the Gel/DEX-Gel was injected submucosally under the direct vision of a 1.2-mm-diameter OCHTA10 nasal endoscope (Additional file 1: Fig. S2). All rats were sacrificed on day 35.

### Diff-quick straining

After sacrifice, the nasal cavity was gently perfused with 1 mL of ice-cold PBS from the trachea to the nasopharynx, and the nasal lavage fluid (NALF) was immediately collected. To evaluate the differential cell counts, 100 µL of NALF was centrifuged using a cytospin (Beyotime Biotechnology, China). The slides were stained with Diff quick (Sysmex Co, Japan) according to the manufacturer's protocol.

### Enzyme-linked immunosorbent assay (ELISA)

The supernatant was collected as previously described. The levels of cytokine IgE, OVA-specific IgE, IL4, IL5, IL13, and IFN-γ in the rats were measured using ELISA kits (Elabscience Biotechnology, China) according to the manufacturer's instructions. The absorbance wavelength of A450nm was measured using a microplate reader, a standard curve was constructed (Additional file 1: Fig. S3), and the concentration value of each sample was calculated.

### Reverse transcription quantitative real-time polymerase chain reaction (RT-qPCR)

Total RNA from cells and the nasal mucosa of rats were extracted using TRIzol (Thermo Fisher, USA) according to the manufacturer's instructions. The RNA was then reverse-transcribed into complementary DNA (cDNA) using the miScript II RT Kit (Takara, Japan). RT-qPCR was performed using the miScript SYBR Green PCR kit (Novoprotein Scientific, China) with the QuantStudio7 Flex PCR system for amplification. The experiment was repeated in triplicate, and the primer sequences are listed in Additional file 1: Fig. S4. Gene expression levels were measured by the 2^−ΔΔCT^ method.

### Hematoxylin–eosin (HE), Toluidine blue (TB), Periodic acid-Schiff (PAS) staining and Immunohistochemistry (IHC)

Following NALF collection, the heads of rats were fixed in 10% neutral buffered formaldehyde solution (Sigma-Aldrich, USA) for two days, decalcified in 0.1 M EDTA buffer (Bio-solution, Korea) for a month, and embedded in paraffin. The blocks were coronally sectioned into 5 µm slices, and some of them were stained with HE (Sigma-Aldrich, USA), PAS (Sigma-Aldrich, USA), and TB (Sigma-Aldrich, USA), respectively.

The other sections were first incubated with anti-GATA-3 antibody 1:200 (Abbiotec-From Biology to Discovery, USA) and anti-T-bet antibody 1:200 (LifeSpan BioSciences, USA) and then left with the secondary antibody in antibody dilution buffer for 1 h at room temperature, rinsed with PBS several times, and DAB was added for color development. Finally, nuclei were counterstained with hematoxylin and assessed by calculating the proportion of positive cells among all or 100 submucosal cells in each high magnification field of view.

The slides were scanned with slide digital scanners Pannoramic DESK (NANOZOOMER S360, China) and examined using Pannoramic case software (NANOZOOMER S360, China). Submucosal eosinophil, mast cell, and goblets counts, as well as positive cells and staining intensity in IHC were performed by 2 pathologists with no prior information about the samples in 10 randomized high magnification fields of view (× 400) in the submucosa of each sample section.

### Flow cytometry analysis

After asphyxiation by carbon dioxide in rats, 20 mg of mucosa was weighed and added to 5 ml of DMEM solution (Biowest, France) containing 5 mg of collagenase type II (Sigma-Aldrich, USA) and 50 µg of DNAase (Sigma-Aldrich, USA) and digested at 37 °C for 1.5 h. The resulting suspension was filtered and centrifuged at 450 g for 5 min. Nasal mucosal cells were obtained by filtration and centrifugation at 450 g for 5 min and resuspended in appropriate PBS. The nasal mucosal cell suspension was divided into several portions, some of which were incubated with IL4-PE (Biolegend, USA), IL5-APC (Biolegend, USA), IL17-AF488 (Biolegend, USA), and IFNγ-PE (Biolegend, USA) at 4 °C for 60 min in the darkness. Another portion of mucosa was incubated with CD4-BV650 (Biolegend, USA) and IL4-PE (Biolegend, USA) or IFNγ-PE (Biolegend, USA) for 60 min at 4 °C in the dark. After rinsing with PBS and resuspension, mucosal samples were transferred to a flow cytomete (FACS Celesta, USA) and analyzed with FlowJo software (version 10.6.2, Treestar, USA).

### Statistical analysis

The experiments were repeated at least three times in each group, and all data were expressed as means ± SD. SPSS software (version 19.0, USA) was used for statistical analysis. Data from different groups were compared by one-way ANOVA. A p < 0.05 was statistically significant.

## Results and discussion

### Preparation and optimization of DEX-Gel

**Fig. 1:**
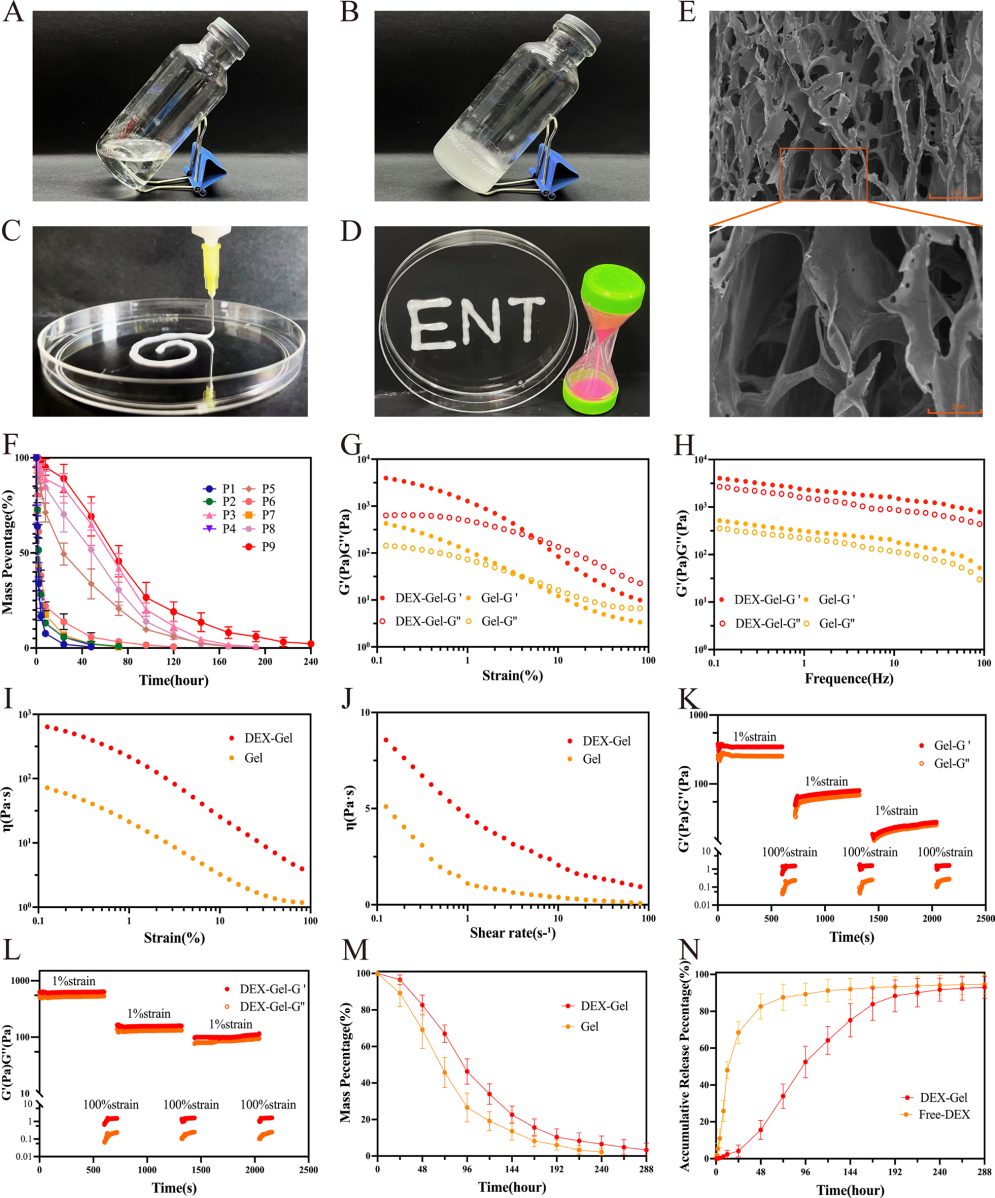
Morphology and characterization of the gels. **A**, **B** Photograph of DEX-Gel after water bath heating and cooling. **C** Photograph of extrusion of injectable Hydrogel by syringe needle Φ = 1.0 mm. **D** Photographs of DEX-Gel sticking vertically to the petri dish. **E** SEM results of DEX-Gel. Scale bars = 100 µm (top) and 35 µm (bottom). **F** Degeneration test of nine prescription gels in vitro. **G** Strain sweep measurements of the storage modulus (G′ denotes the elastic modulus, and G′′ denotes the loss modulus). The frequency was 1 Hz. **H** Frequency sweep measurements of the storage modulus. The strain was 1%. **I** Strain sweep measurements of the viscosity parameters. The frequency was 1 Hz. **J** Shear rate sweep measurements of the viscosity parameters. The frequency was 1 Hz. **K**, **L** Measure of storage modulus in relation to time in seconds. The strain shearing rate alternated between 1% strain for 600 s and 100% strain for 120 s. **M** Degeneration test of Gel and DEX-Gel in vitro. **N** Drug release of DEX-Gel ang Free-DEX in vitro

Injectable gels were formulated by blending Pluronic F127, SA, and PEG400, followed by heating in a water bath. After heating, the three components were thoroughly mixed, resulting in a homogeneous, transparent-like liquid (Fig. 1A), which could be solidified into a milky-white, homogeneous gel upon cooling at room temperature (Fig. 1B). In this study, a two-factor, three-level orthogonal experiment was conducted on the composition ratio, considering the dissolution time of various prescription gels and the maximum loading of DEX (Additional file 1: Fig. S5). The results indicated that the dissolution time of the P9 prescription reached 14 days (Fig. 1F), with a maximum drug loading capacity of 30 mg/ml. Furthermore, Nuclear Magnetic Resonance (NMR) confirmed the presence of active groups in DEX dissolved within DEX-Gel (Additional file 1: Fig. S6).

### Characterization of DEX-Gel

Extrusion experiments demonstrated that DEX-Gel exhibited sufficient mechanical strength to smoothly pass through a Φ1.0 mm injection needle, forming a three-dimensional gel (Fig. 1C). Additionally, when placed vertically on a petri dish, DEX-Gel adhered tightly, maintaining its intact morphology, showcasing its favorable viscosity (Fig. 1D). Scanning electron microscopy (SEM) showed that the freeze-dried DEX-Gel had a lamellar pore structure. The loading of drug molecules due to the hydrophobic interaction between DEX-Gel components. To assess the mechanical properties of the gels, rheological analysis was conducted on Gel and DEX-Gel. Both gels remained in a solid state at stresses less than 1%, with the storage modulus (G') consistently exceeding the loss modulus (G"). Notably, the G' of DEX-Gel (≈4000 Pa) significantly surpassed that of Gel (≈500 Pa)(Fig. 1G). At frequencies below 100 Hz, both gels maintained a solid state, with DEX-Gel exhibiting a higher G' (≈4200 Pa) compared to Gel (≈550 Pa) (Fig. 1H).

During injection through a syringe, DEX-Gel initially exhibited shear thinning but regained its complete form after injection cessation, demonstrating a "self-healing" ability crucial for intranasal gel injection [24–26]. Dynamic stress changes confirmed the rapid recovery of both Gel and DEX-Gel modulus when subjected to a 100% to 1% stress transition, with DEX-Gel exhibiting a higher degree of recovery (Fig. 1K, L). Viscosity experiments under different shear stresses and shear rates consistently revealed higher viscosity for DEX-Gel compared to Gel (Fig. 1I, J).

The above experiments indicated that the mechanical strength and viscosity of the gels were enhanced upon DEX encapsulation, potentially due to increased crosslink density. Moreover, repeated alternations of shear stress confirmed the gradual recovery of the DEX-Gel storage modulus, affirming its stable solid structure and validating its injectability and self-healing properties.

Before evaluating the drug release ability of the gels, we first examined the degradation characteristics of Gel and DEX-Gel. As depicted in Fig. 1M, in the in vitro assay, Gel experienced degradation by day 10, while DEX-Gel retained 6.51 ± 3.62% of its initial mass. And in the in vivo degradation test, DEX-Gel was degraded on day 18 (Additional file 1: Fig. S7). In subsequent drug release experiments, the Free-DEX group exhibited a release rate of 68.5 ± 4.94% at 24 h, escalating to 87.53 ± 5.83% at 72 h, with a final release rate of 94.69 ± 4.28%. Conversely, DEX-Gel demonstrated a release rate of only 4.12 ± 2.64% at 24 h, reaching 33.91 ± 5.41% at 72 h. The final release rate for DEX-Gel was 93.01 ± 4.87%. Notably, compared to Free-DEX, DEX-Gel exhibited a stable drug release profile over the entire 14-day period (Fig. 1N).

Pluronic F127 is commonly employed in the formulation of temperature-sensitive hydrogels [27–29]. While Pluronic F127 gels alone exhibited low stability, the addition of SA and PEG400 could mitigate the adverse effects associated with sudden gel disintegration [30, 31]. This combination contributed to the stabilization of DEX-Gel, facilitating a controlled and sustained release mechanism.

### DEX-Gel exhibited good biocompatibility

**Fig. 2:**
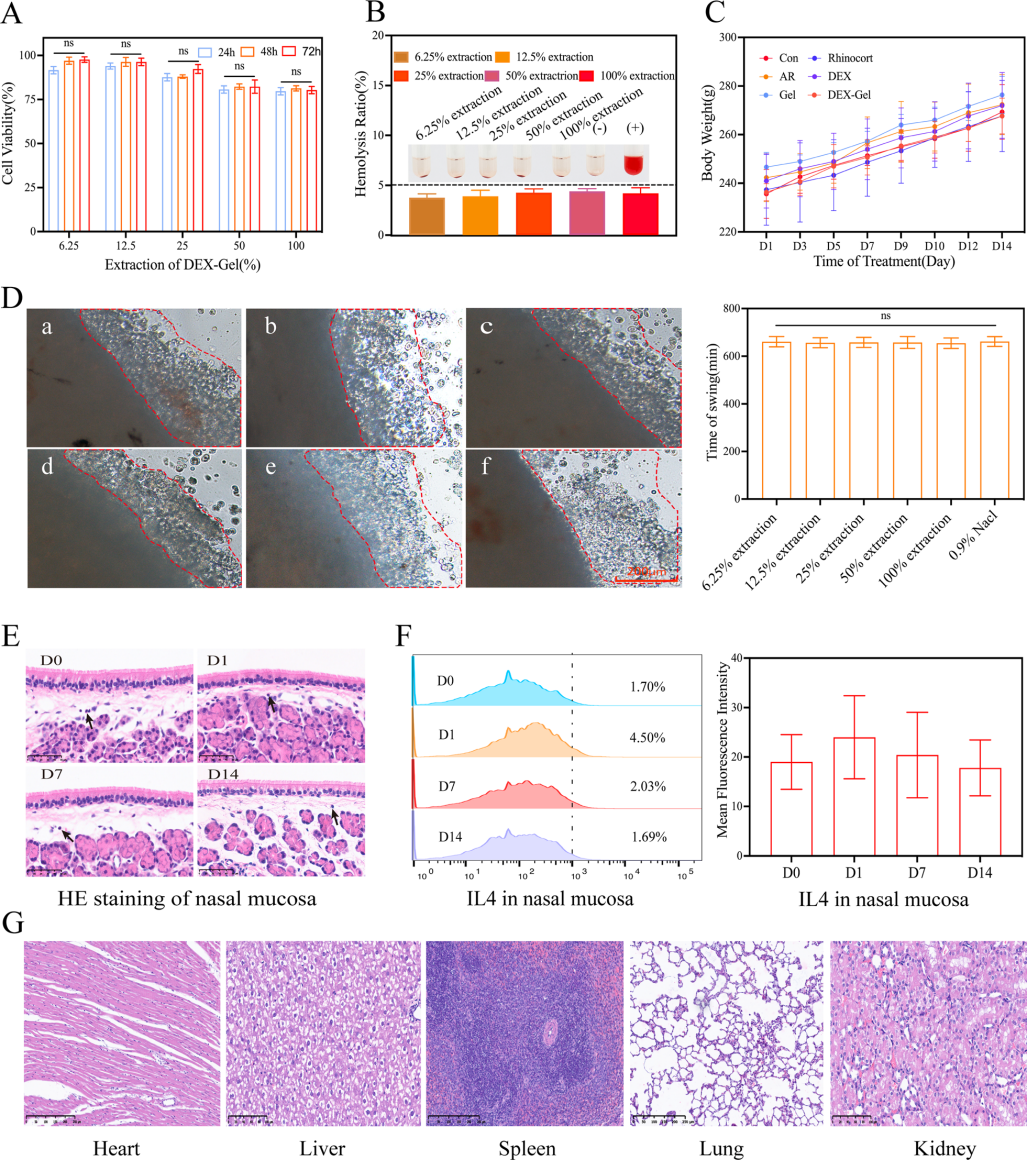
DEX-Gel has superior biocompatibility. **A** Viability of the L929 cells in different concentrations of DEX-Gel extract for 24, 48, and 72 h (n = 3). **B** The hemolysis ratio in different concentrations of DEX-Gel extraction、PBS (−) and distilled water ( +) (n = 3). **C** The body weight of rats in different groups (n = 5). **D** Morphology and oscillation duration of the maxillary mucosa (red marker area) of *Bufo chinensis* in different concentrations of DEX-Gel extracts. **a** PBS, **b**–**f** 6.25%, 12.5%, 25%, 50%, and 100% concentration of DEX-Gel extract. **E**, **F** Photographs of HE staining and IL4 levels in the nasal mucosa. Arrows point to inflammatory cells (n = 3). **G** HE staining Photographs of major organs. Scare bars = 200 µm in (**D**); Scare bars = 50 µm in (**E**); Scare bars = 250 µm in (**G**). Data are expressed as mean ± SD. *P < 0.05, **P < 0.01 and ***P < 0.001, significantly different from the ANOVA group

To evaluate the biocompatibility of DEX-Gel, we initially employed CCK-8 assays to assess its cytotoxicity on L929 cells. Figure 2A illustrates that approximately 80% of cells survived on days 1, 2, and 3 at various concentrations of DEX-Gel extracts, indicating the non-toxic nature of DEX-Gel to L929 cells. Given potential gel infiltration into blood vessels during submucosal injection, we further investigated the hemolysis rate of DEX-Gel. As depicted in Fig. 2B, the hemolysis rate of different concentrations of DEX-Gel extracts was below 5%, substantiating the absence of a significant hemolytic reaction.

It is well-established that the nasal cavity's mucosal ciliary clearance, a crucial element of innate immunity, involves the mucus layer, airway surface fluid layer, and ciliated epithelium [32, 33]. To assess the impact of DEX-Gel on mucosal cilia in *Bufo chinensis*, we observed the effects of DEX-Gel on ciliary movement in the palate. As shown in Fig. 2D, cilia in various concentrations of DEX-Gel extract were well-organized, exhibiting orderly swinging without a significant difference in ciliary movement duration compared to the PBS group.

Following biomaterial implantation, tissue damage can trigger a foreign body reaction (FBR). Monitoring FBR involves assessing inflammation through HE staining and IL4 levels [34–36]. In this study, HE staining of nasal mucosa after DEX-Gel injection on days 0, 1, 7, and 14, along with IL4 level examination, revealed no significant increase in inflammatory cells. While the IL4 level increased after the first day of DEX-Gel injection, the change was not statistically significant, suggesting that the submucosal injection of DEX-Gel induces either minimal or no foreign body reaction.

In addition to this, we closely monitored the body weight of the rats throughout the treatment period and conducted HE staining on major organs. The findings revealed a gradual increase in the body weight of rats across all groups, with no significant differences observed. Importantly, no signs of toxic damage to major organs were observed in any group (Fig. 2G, Additional file 1: Fig. S8).

The above results substantiate the excellent biosafety and histocompatibility of DEX-Gel, attributed to the fact that all components used in DEX-Gel were FDA-approved materials [37–39]. Most importantly, the preparation process of DEX-Gel avoids the use of any toxic reagents, further contributing to its safety profile.

### Establishment of an allergic rat model by OVA nasal drip and preliminary evaluation of DEX-Gel treatment effect by detecting mRNA expression of inflammatory factors

**Fig. 3:**
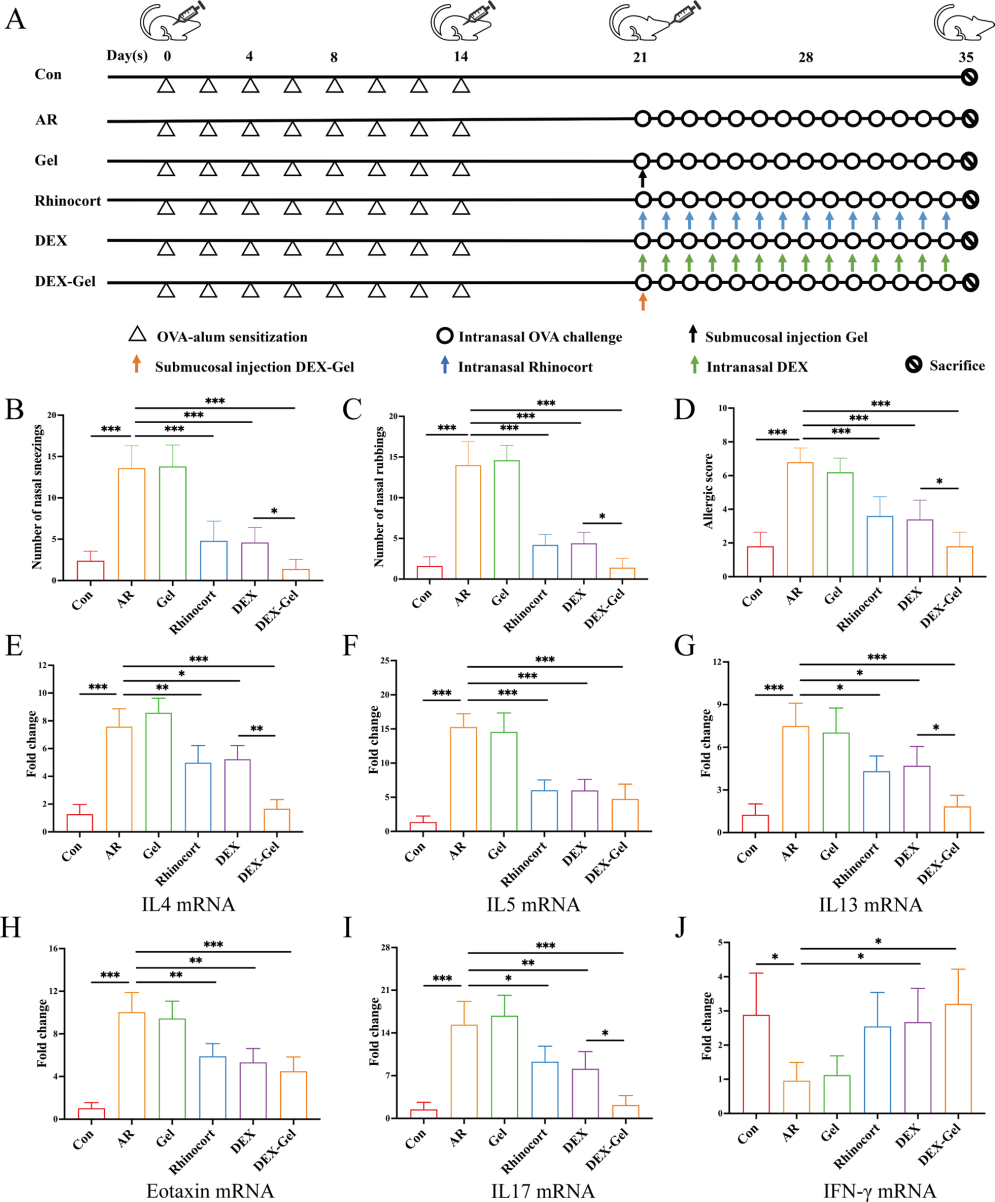
Schematic diagram of OVA-induced AR rat model and DEX-Gel inhibited mRNA expression of inflammatory cytokines. **A** SD rats were sensitized with 50 µg OVA together with 4 mg Alum (400 µL) by intraperitoneal injection on days 0, 2, 4, 6, 8, 10, 12 and 14. On days 21–34, except for group control, SD rats were stimulated with intranasal drops of 1250 µg/20 µL OVA daily. From day 21 to 34, group Rhinocort or DEX was treated with nasal drops of 50 µg/20 µL Rhinocort or DEX; group AR was challenged with PBS; group Gel or DEX-Gel was treated with nasal submucosa injection of 800 µg/200 µL Gel or DEX-Gel on days 21. All SD rats were sacrificed on day 35. **B**, **C**, **D** The number of nasal rubbings, sneezing and allergic scores of rats after treatment (n = 5). **E**–**J** The mRNA expression of different inflammatory factors in the nasal mucosa was determined by RT-qPCR (n = 3). Data are expressed as mean ± SD. *P < 0.05, **P < 0.01 and ***P < 0.001, significantly different from the ANOVA group

To gain a deeper understanding of allergic rhinitis pathophysiology, various attempts to establish animal models have been reported in the literature [40, 41]. In this study, Sprague–Dawley (SD) rats were chosen for their suitability for minimally invasive gel injection. The AR model was induced by intraperitoneal injection and local nasal excitation, following the method described by Dong et al. [42] and Tatar et al. [43] with slight modifications (Fig. 3A). AR was assessed using nasal allergic scores, which included parameters such as nasal rubbing, sneezing, and nasal flow (Additional file 1: Fig. S9). AR development was confirmed if the total scores exceeded 5 points [44]. On day 28, after the last nasal challenge, rats were observed for approximately 15 min, and allergic scores were assessed. The allergic score exceeded 5 points in the AR group.

After 2 weeks of different treatments, the number of nasal rubs and sneezes, along with allergy scores, were recorded (Fig. 3B, C, and D). The allergic symptoms in the Rhinocort group (AstraZeneca AB), DEX group, and DEX-Gel group were alleviated compared to the AR group, with the most pronounced treatment effect observed in the DEX-Gel group.

Cytokine mRNA expression of T_H_1 cytokines (IFN-γ), T_H_2 cytokines (IL4, IL5, and IL13), and other pro-inflammatory cytokines (IL17, Eotaxin) in the nasal mucosa was examined using RT-qPCR (Fig. 3E–J). All cytokines, except IFN-γ, were upregulated in the AR group. The Rhinocort, DEX, and DEX-Gel groups inhibited the upregulated cytokines to varying degrees, consistent with previous findings [45–47]. No significant differences in the levels of inflammatory mediator were observed between the Gel and AR groups, indicating that submucosal injections of Gel did not induce additional inflammatory responses. In terms of inhibiting IL5 and Eotaxin, there was no significant difference between the DEX-Gel, Rhinocort, and DEX groups. However, DEX-Gel exhibited a more significant inhibition of IL4 and IL13, which may be attributed to the fact that IL4 and IL13 are primary sensitizing factors in type I allergic reactions, being secreted in large quantities during T_H_2-type immune responses.

### DEX-Gel alleviates nasal mucosal edema by reducing submucosal inflammatory cell infiltration

**Fig. 4:**
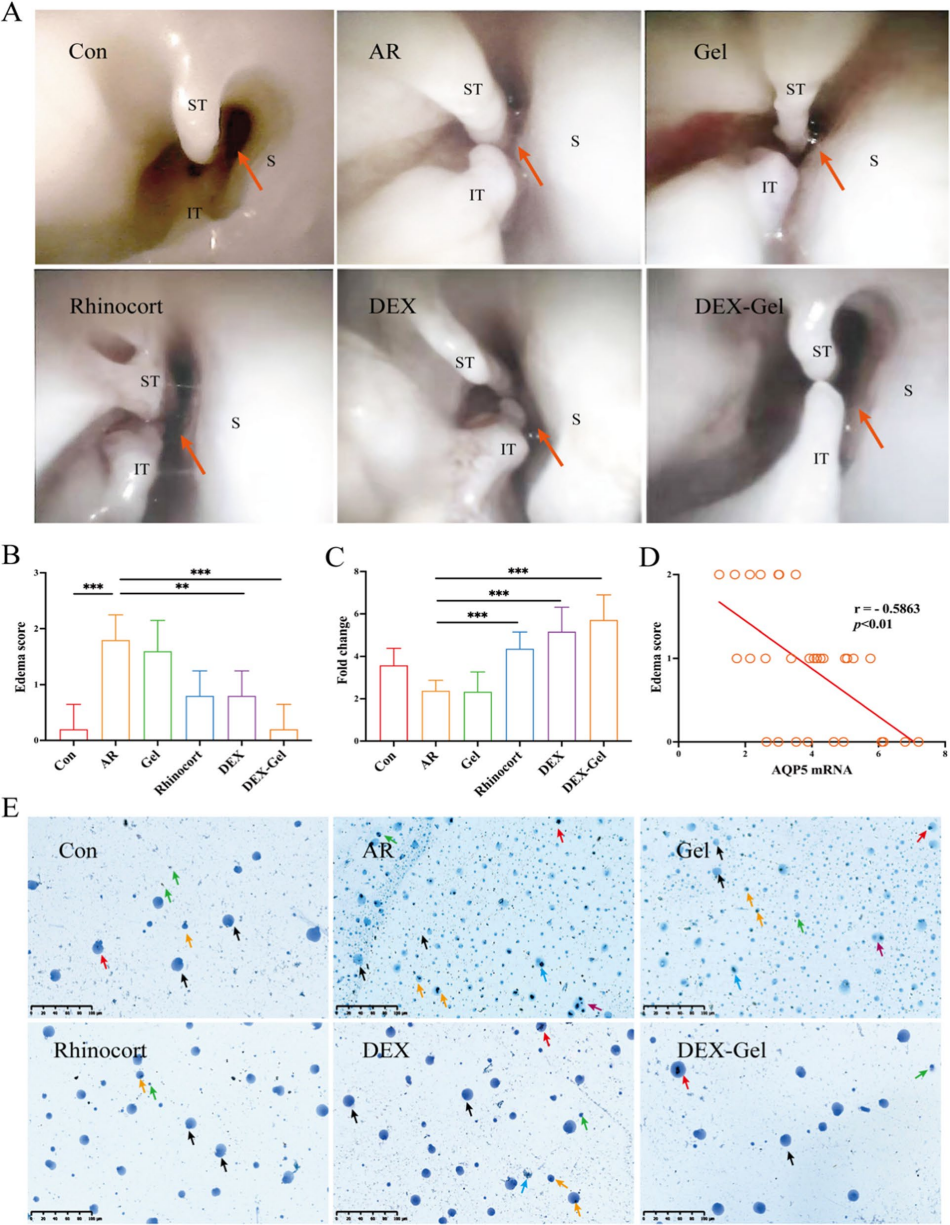
DEX-Gel alleviates the edema of the turbinate mucosa and inhibits differentiated inflammatory cells in NALF. **A** Representative endoscopic images of the rat nasal cavity. Arrows point to meatus nasi communis (nasal passages for breathing). ST: superior turbinate; IT: inferior turbinate; S: septum. **B** The edema score of rats (n = 5). **C** The mRNA expression of AQP5 (n = 5). **D** Correlation analysis between edema score and expression of AQP5 mRNA (n = 30). **E** Representative Diff quick stained images of the NALF. Black arrows, red arrows, yellow arrows, green arrows, blue arrows and purple arrows represent epithelial cells, macrophages, eosinophils, neutrophils, lymphocytes and mast cells, respectively. Scale bars = 100 µm. Data are expressed as mean ± SD. *P < 0.05, **P < 0.01 and ***P < 0.001, significantly different from the ANOVA group

Allergic rhinitis manifests in nasal endoscopy as pale and edematous turbinates. Edema was semiquantitatively evaluated based on the scoring system by Zhang et al. [48], where a score of 0 indicated no edema, 1 indicated mild/moderate edema, and 2 indicated intense edema. Rhinoscopy (Fig. 4A) and edema scoring (Fig. 4B) were performed on all rats. Treatment with Rhinocort or DEX partially reduced turbinate swelling in SD rats, leading to relatively patent nasal passages. In contrast, treatment with DEX-Gel significantly reduced turbinate swelling in SD rats, resulting in partially cleared nasal passages.

To provide an objective assessment of edema, we analyzed AQP5 mRNA expression, given its crucial role in maintaining mucosal water homeostasis and alleviating mucosal edema [49]. The mRNA expression of AQP5 in the DEX-Gel group was significantly higher than that in the AR group (Fig. 4C). Additionally, we assessed the correlation between the edema score and AQP5 mRNA expression, revealing a negative correlation (Fig. 4D). Although the decline in AQP5 mRNA expression in the AR group compared to the control group was not statistically significant (P = 0.051), this phenomenon aligns with findings in chronic rhinosinusitis reported by Gao et al. [50]. The shift in AQP5 expression from the apical portion of the surface epithelium to the cytoplasm of the glandular epithelium may explain this trend [51]. Further studies are warranted to confirm the protein expression pattern of AQP5 in AR.

Cell differentiation in NALF obtained (Fig. 4E) was observed through Diff-Quick staining. The group AR exhibited a high number of differentiated cells, including epithelial cells, eosinophils, neutrophils, lymphocytes, mast cells, and macrophages (Additional file 1: Fig. S10). Both Rhinocort and DEX significantly reduced the number of epithelial cells in NALF, along with a reduction in eosinophils and macrophages. The DEX-Gel group, on the other hand, reduced the abundance of differentiated cells, including lymphocytes and mast cells, despite their small cellular volume, which could be attributed to the fact that epithelial cells were the first to encounter environmental triggers, such as pathogens and allergens. Following treatment, there was a reduction in inflammatory infiltrate, and the activity of epithelial cells significantly decreased. Consequently, a decrease in the percentage of epithelial cells was observed in the Rhinocort, DEX, and DEX-Gel groups. In terms of neutrophils, lymphocytes, and mast cells, no notable differences were found after treatment with Rhinocort or DEX, as their numbers were too low in the NALF. These parameters were only altered in the DEX-Gel group, which is characterized by long-lasting slow-release anti-inflammatory effects.

**Fig. 5:**
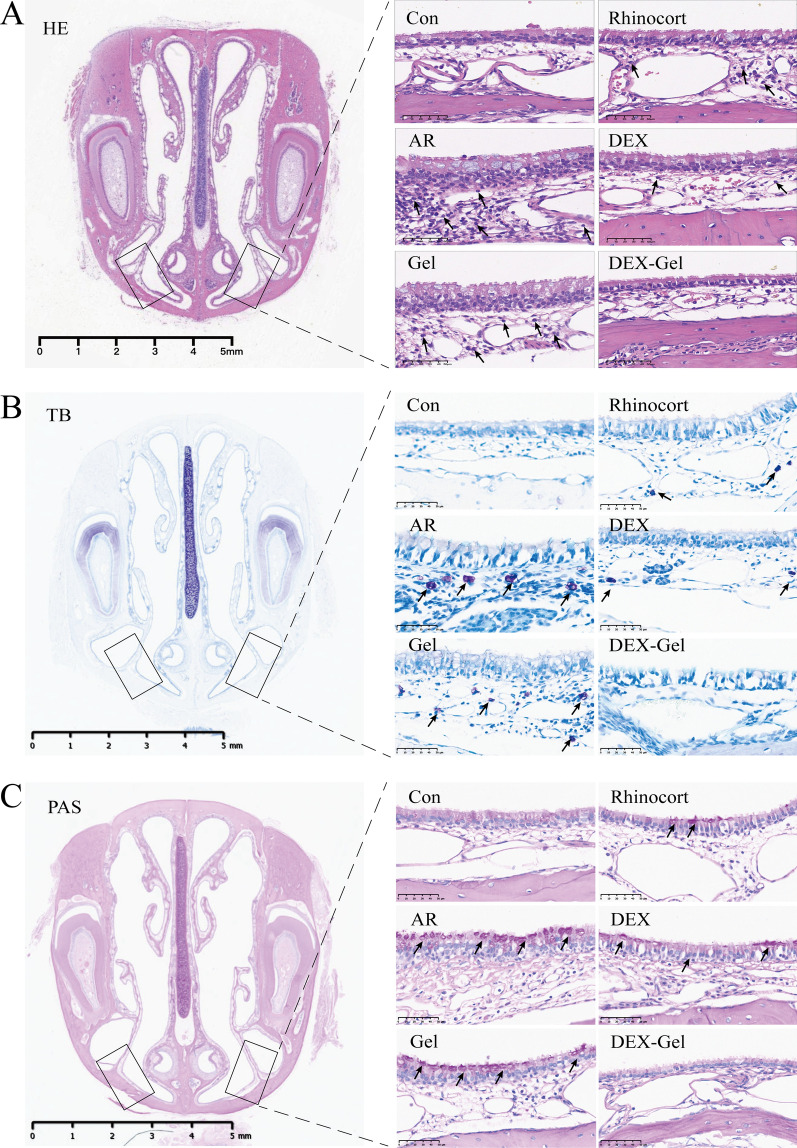
DEX-Gel reversed the effects of OVA-induced histopathological changes on nasal mucosa. **A** Representative HE-stained images of nasal mucosal thickness and eosinophils. Arrows point to eosinophils. **B** Representative TB-stained images of mast cells. Arrows point to mast cells stained purple. **C** Representative PAS-stained images of goblet cells. Arrows point to goblet cells stained purple. Scale bars = 5 mm on the left side of (**A**, **B**, **C**); Scale bars = 50 µm on the right side of (**A**, **B**, **C**). Data are expressed as mean ± SD. *P < 0.05, **P < 0.01 and ***P < 0.001, significantly different from the ANOVA group

Allergic rhinitis symptoms predominantly manifest in the nose [52]. We monitored nasal histopathological changes in the nasal tissue sections of rats using HE staining. The AR group exhibited increased thickness due to the accumulation of inflammatory cells beneath the epithelium (Additional file 1: Fig. S11). Eosinophils, stained red in HE staining, showed concentrated infiltration in areas with confirmed nasal histopathological changes. Treatment with Rhinocort, DEX, and DEX-Gel alleviated these conditions (Fig. 5A, Additional file 1: Fig. S11), consistent with the decrease in eosinophil numbers in NALF following DEX-Gel treatment (Additional file 1: Fig. S10).

To identify the cell type contributing to the increased nasal tissue thickness, we stained mast cells (Fig. 5B) using Toluidine Blue. Mast cells, stained in purple, exhibited significantly elevated abundance in the AR group compared to the control group. Group DEX-Gel exhibited a significant decrease in mast cell numbers compared to the AR group, and no degranulation or histamine release was observed.

Hypertrophic changes in goblet cells, associated with mucus secretion, are a significant feature of respiratory epithelial inflammation [53]. Therefore, we analyzed goblet cell hyperplasia in nasal tissue sections using PAS staining. The number of goblet cells dramatically increased in the AR group but significantly decreased in the DEX-Gel group (Fig. 5C).

Taken together, our findings suggest that DEX-Gel therapy effectively alleviated allergic symptoms by controlling the infiltration of inflammatory.

cells in the nasal mucosa, surpassing the outcomes observed in other treatment groups.

### DEX-Gel inhibits overactivated T_H_2 immune responses and promotes the rebalance of T_H_1/T_H_2 in nasal mucosa

In the context of allergic rhinitis, the imbalance of T_H_1/T_H_2 immune responses is recognized as a pivotal factor [12, 54]. T_H_1 cells produce IFN-γ to inhibit T_H_2 cells, and reciprocally, T_H_2 cells produce IL4 to inhibit T_H_1 cells. Current evidence suggests that this dynamic balance is essential for maintaining the body's health. Upon initial exposure to an allergen, inhaled antigens are transported by dendritic cells to lymph nodes or local mucosa.

Activated dendritic cells present allergen-derived peptides to naive T cells, prompting their differentiation into T_H_2 cells. As the number of T_H_2 cells increase, the feedback regulation by T_H_1 cells is weakened, disrupting the T_H_1/T_H_2 balance. Over-activated T_H_2 cells release abundant IL4 and IL13, stimulating B cells to produce IgE. IgE bound to and sensitized tissue-resident mast cells, leading to immediate allergic reactions upon re-exposure to the allergen [55–57].

**Fig. 6:**
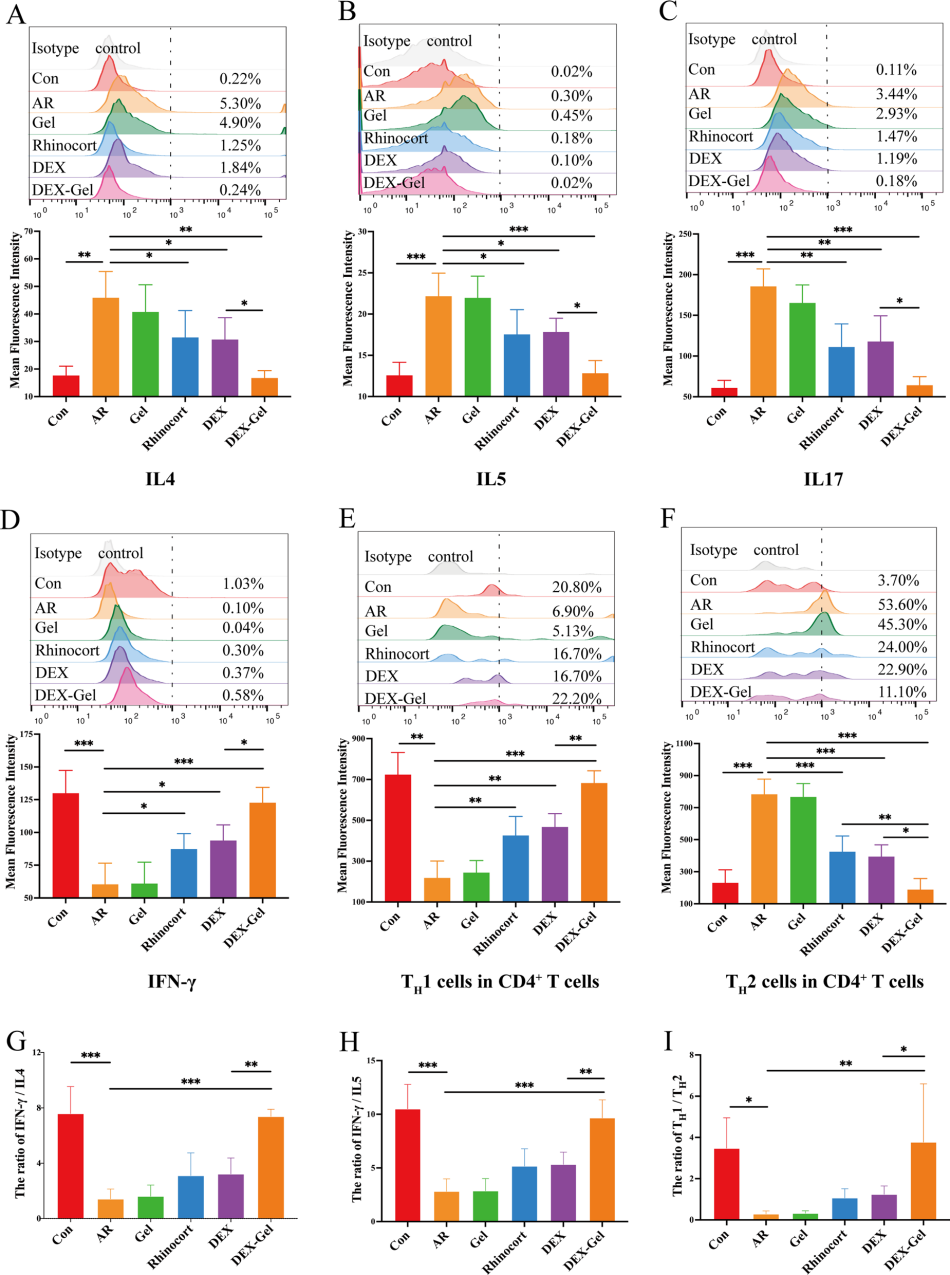
DEX-Gel regulates the inflammatory cytokines and the balance of T_H_I/T_H_2 cell subsets. **A**–**F** Representative flow charts and changes in the numbers of IL4, IL5, IL17, IFN-γ, CD4^+^ IFN-γ^+^ T_H_1, and CD4^+^ IL4^+^ T_H_2 cell subsets in the nasal mucosa (n = 3). **G**–**I** The ratio of IFN-γ/IL4, IFN-γ/IL5, and T_H_1/T_H_2 cell subsets in the nasal mucosa (n = 3). Data are expressed as mean ± SD. *P < 0.05, **P < 0.01 and ***P < 0.001, significantly different from the ANOVA group

To investigate the impact of DEX-Gel on nasal mucosa immune responses, we examined the levels of inflammatory mediators (IL4, IL5, IFN-γ, IL17) and the proportions of T_H_1 and T_H_2 cells using flow cytometry (Fig. 6A–F). OVA increased the percentage of IL4 and IL5 while decreasing IFN-γ, indicating overactivation of T_H_2 responses. Notably, DEX-Gel reversed these OVA-induced changes. Both Rhinocort and DEX groups suppressed T_H_2-type inflammatory mediators and partially elevated T_H_1-type inflammatory mediators, suggesting a potential for rebalancing T_H_1/T_H_2 in AR. However, a comprehensive comparison using IFN-γ/IL4, IFN-γ/IL5, and the T_H_1/T_H_2 cells in CD4^+^ T cells ratio revealed that only the DEX-Gel group effectively reversed the immune imbalance (Fig. 6G–I). Based on our findings, it is highly conceivable that the sustained slow release of DEX-Gel over 2 weeks played a crucial role in amplifying DEX's ability to suppress T_H_2 responses and enhance T_H_1 responses, ultimately achieving the regulation of the T_H_1/T_H_2 balance.

**Fig. 7:**
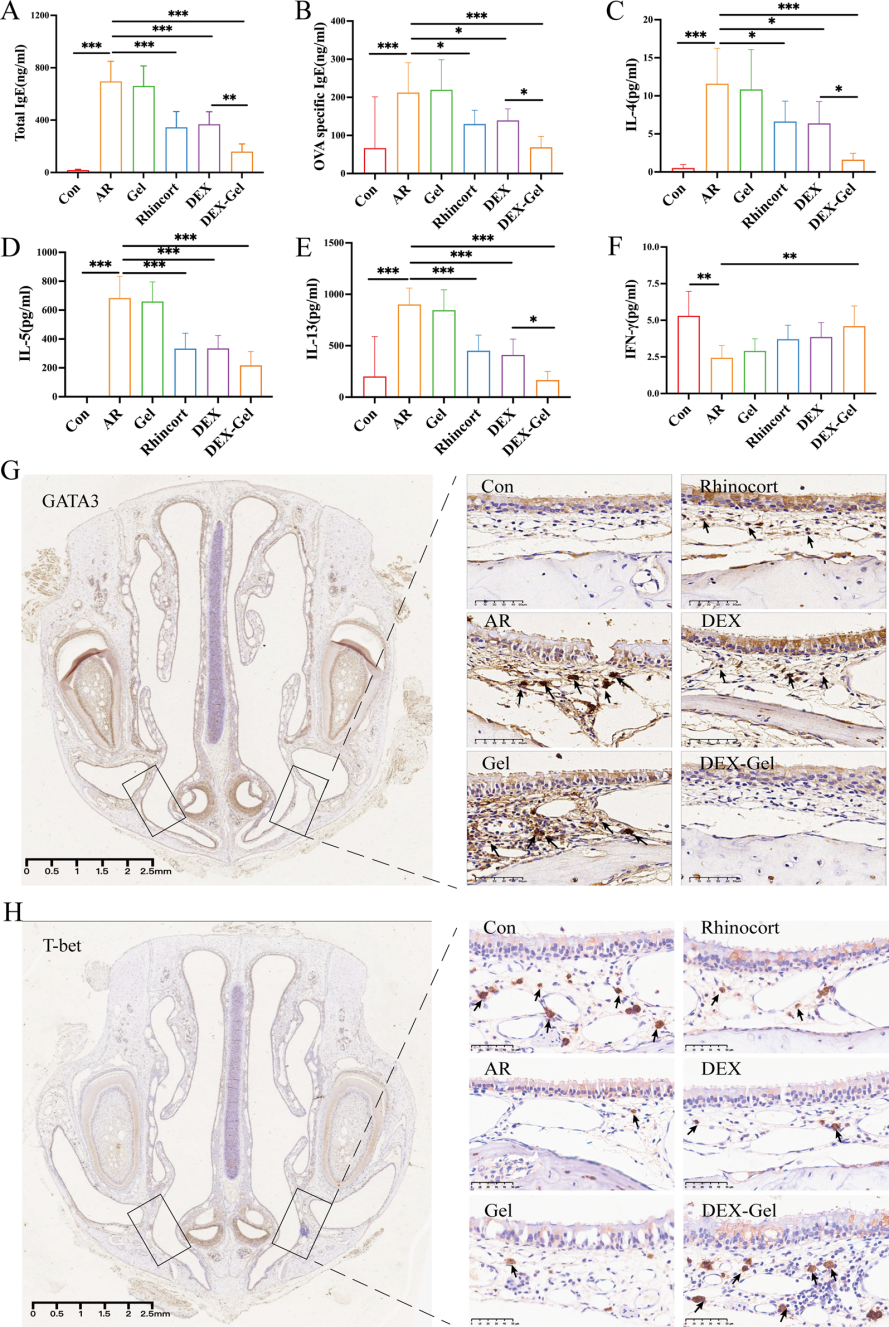
DEX-Gel regulates the expression of inflammatory factors in NALF as well as GATA-3 and T-bet in histopathological tissues. **A**–**F** Total IgE, OVA-Specific IgE, IL4, IL5, IL13, and IFN-γ levels in the NALF were quantified by ELISA (n = 3). **G** Representative IHC images of the GATA-3^+^ cells. Arrows point to nuclear-stained GATA-3^+^ cells. **H** Representative IHC images of the T-bet^+^ cells. Arrows point to nuclear-stained T-bet^+^ cells. Scale bars = 2.5 mm on the left side of (**G**, **H**); Scale bars = 50 µm on the right side of (**G**, **H**). Data are expressed as mean ± SD. *P < 0.05, **P < 0.01 and ***P < 0.001, significantly different from the ANOVA group

In addition to flow cytometry, various inflammatory mediators, including IgE and OVA-specific IgE, were measured in NALF using ELISA. In the OVA-induced AR group, the levels of total IgE (Fig. 7A) and OVA-specific IgE (Fig. 7B) were significantly elevated in NALF. Remarkably, DEX-Gel demonstrated significant inhibition of both total IgE and OVA-specific IgE. Furthermore, OVA increased the levels of IL4, IL5, and IL13 while decreasing the levels of IFN-γ. DEX-Gel effectively reversed these OVA-induced effects (Fig. 7C–F), consistent with the findings from the flow cytometry assay.

GATA-3, recognized as the T_H_2 transcription factor, plays a crucial role in regulating T_H_2 cytokine expression by binding to the promoters of IL5.

and IL13 genes and is involved in chromatin remodeling to open the IL4 locus [58, 59]. GATA-3 can also repress T_H_1 cytokine production through antagonism with IL12 [60]. On the other hand, T-bet, a regulator of T_H_1 cells, binds to and promotes repressive chromatin modifications at the GATA-3 locus, inhibiting GATA-3 expression in T_H_1 cells [61]. The deficiency of T-bet in mice results in airway eosinophilia, overexpression of T_H_2 cytokines, and airway hyper-responsiveness [62]. IHC staining of the nasal mucosa was performed to assess the expression of T-bet and GATA-3. The results, shown in Fig. 7G, H, revealed GATA-3 overexpression in the AR group compared with the control group, while T-bet expression was significantly reduced. Rhinocort or DEX treatment led to some changes in T-bet expression, but no significant difference was observed compared with the AR group (Additional file 1: Fig. S11). A complete reversal of the changes induced by OVA in GATA-3 and T-bet expression was observed only in the DEX-Gel group (Additional file 1: Fig. S12).

## Conclusion

Herein, we devised an easily synthesized injectable DEX-Gel with a sustained release duration of 14 days for the treatment of AR. In vitro experiments demonstrated the favorable physical and biochemical properties of DEX-Gel, including injectability, biodegradability, stable drug release, and some self-healing capabilities. In vivo experiments revealed that DEX-Gel effectively suppressed T_H_2-type inflammatory factors such as IL4, IL5, and IL13. Moreover, it ameliorated the alterations in GATA-3 and T-bet induced by OVA, ultimately restoring the T_H_1/T_H_2 immune balance. Notably, the application of DEX-Gel exhibited significant anti-inflammatory effects without causing significant side effects. Overall, our experimental findings suggest that our gel represents an ideal drug carrier for locally administered minimally invasive treatment of AR, ensuring safety and reliability. Indeed, the precise administration by a specialist for varied conditions minimizes the risk of under- or over-medication, providing a flexible and tailored strategy for clinical treatment.

### Supplementary Information

**Additional file 1: S1.** Standard curve for dexamethasone (DEX) in drug release assays. **S2.** Schematic diagram of endoscopic mucosal injection of DEX-Gel, with the arrow pointing to the injection needle. **S3.** Standard curve of total IgE (A), OVA-specific IgE (B), IL4 (C), IL5 (D), IL13 (E) and IFN-γ (F) in ELISA assays. **S4.** Primer sequences for different genes. Gene expression levels were measured by the 2-ΔΔCT method. **S5.** Two-factor, three-level orthogonal experiment, P9 prescription maximum loadable dexamethasone 30 mg. **S6.** Molecular structure of DEX, with the active groups shown in the black rectangle(A).NMR detection of Free DEX and DEX-Gel, with the active groups shown in the black rectangle(B). **S7.** DEX-Gel in vitro degradation experiments, where yellow arrows show DEX-Gel. **S8.** HE staining of the main organs of SD rats in the groups Con、AR、Gel、Rhinocort and DEX. Scare bars = 250 µm. **S9.** Allergy score in rat model of AR, total score > 5 considered successful modeling. **S10.** Number of different cells in nasal lavage fluid. Data are expressed as mean ± SD. *P < 0.05, **P < 0.01 and ***P < 0.001, significantly different from the ANOVA group. **S11.** Mucosal thickness (A), number of eosinophils (B), mast cells (C) and goblet cells (D) in groups Con, AR, Gel, Rhinocort, DEX and DEX-Gel. Data are expressed as mean ± SD. *P < 0.05, **P < 0.01 and ***P < 0.001, significantly different from the ANOVA group. **S12.** Immunohistochemical scores for GATA-3 (A) and T-bet (B) in groups Con, AR, Gel, Rhinocort, DEX and DEX-Gel. The scoring system included assigning scores based on the percentage of positive cells: 0 (< 5%), 1 (5–25%), 2 (25–50%), 3 (50–75%), and 4 (> 75%). Staining intensity was also scored: 0 (colorless), 1 (light yellow), 2 (tan), and 3 (brown). The total score was determined by multiplying the positive cell score with the staining intensity score. Data are expressed as mean ± SD. *P < 0.05, **P < 0.01 and ***P < 0.001, significantly different from the ANOVA group.

## Data Availability

All data generated and analyzed during this research are included in this published article and its additional file.
