# Supplementary material for: Long-acting anti-inflammatory injectable DEX-Gel with sustained release and self-healing properties regulates TH1/TH2 immune balance for minimally invasive treatment of allergic rhinitis
Source: J Nanobiotechnology. 2024 Feb 6;22:51. doi: 10.1186/s12951-024-02306-w (PMC10845556; doi:10.1186/s12951-024-02306-w)
Supplement: Supplementary file 1 — Additional file 1: S1. Standard curve for dexamethasone (DEX) in drug release assays. S2. Schematic diagram of endoscopic mucosal injection of DEX-Gel, with the arrow pointing to the injection needle. S3. Standard curve of total IgE (A), OVA-specific IgE (B), IL4 (C), IL5 (D), IL13 (E) and IFN-γ (F) in ELISA assays. S4. Primer sequences for different genes. Gene expression levels were measured by the 2-ΔΔCT method. S5. Two-factor, three-level orthogonal experiment, P9 prescription maximum loadable dexamethasone 30 mg. S6. Molecular structure of DEX, with the active groups shown in the black rectangle(A).NMR detection of Free DEX and DEX-Gel, with the active groups shown in the black rectangle(B). S7. DEX-Gel in vitro degradation experiments, where yellow arrows show DEX-Gel. S8. HE staining of the main organs of SD rats in the groups Con、AR、Gel、Rhinocort and DEX. Scare bars = 250 µm. S9. Allergy score in rat model of AR, total score > 5 considered successful modeling. S10. Number of different cells in nasal lavage fluid. Data are expressed as mean ± SD. *P < 0.05, **P < 0.01 and ***P < 0.001, significantly different from the ANOVA group. S11. Mucosal thickness (A), number of eosinophils (B), mast cells (C) and goblet cells (D) in groups Con, AR, Gel, Rhinocort, DEX and DEX-Gel. Data are expressed as mean ± SD. *P < 0.05, **P < 0.01 and ***P < 0.001, significantly different from the ANOVA group. S12. Immunohistochemical scores for GATA-3 (A) and T-bet (B) in groups Con, AR, Gel, Rhinocort, DEX and DEX-Gel. The scoring system included assigning scores based on the percentage of positive cells: 0 (< 5%), 1 (5–25%), 2 (25–50%), 3 (50–75%), and 4 (> 75%). Staining intensity was also scored: 0 (colorless), 1 (light yellow), 2 (tan), and 3 (brown). The total score was determined by multiplying the positive cell score with the staining intensity score. Data are expressed as mean ± SD. *P < 0.05, **P < 0.01 and ***P < 0.001, significantly different from the ANOVA group. [file 12951_2024_2306_MOESM1_ESM.docx]

**S1.**


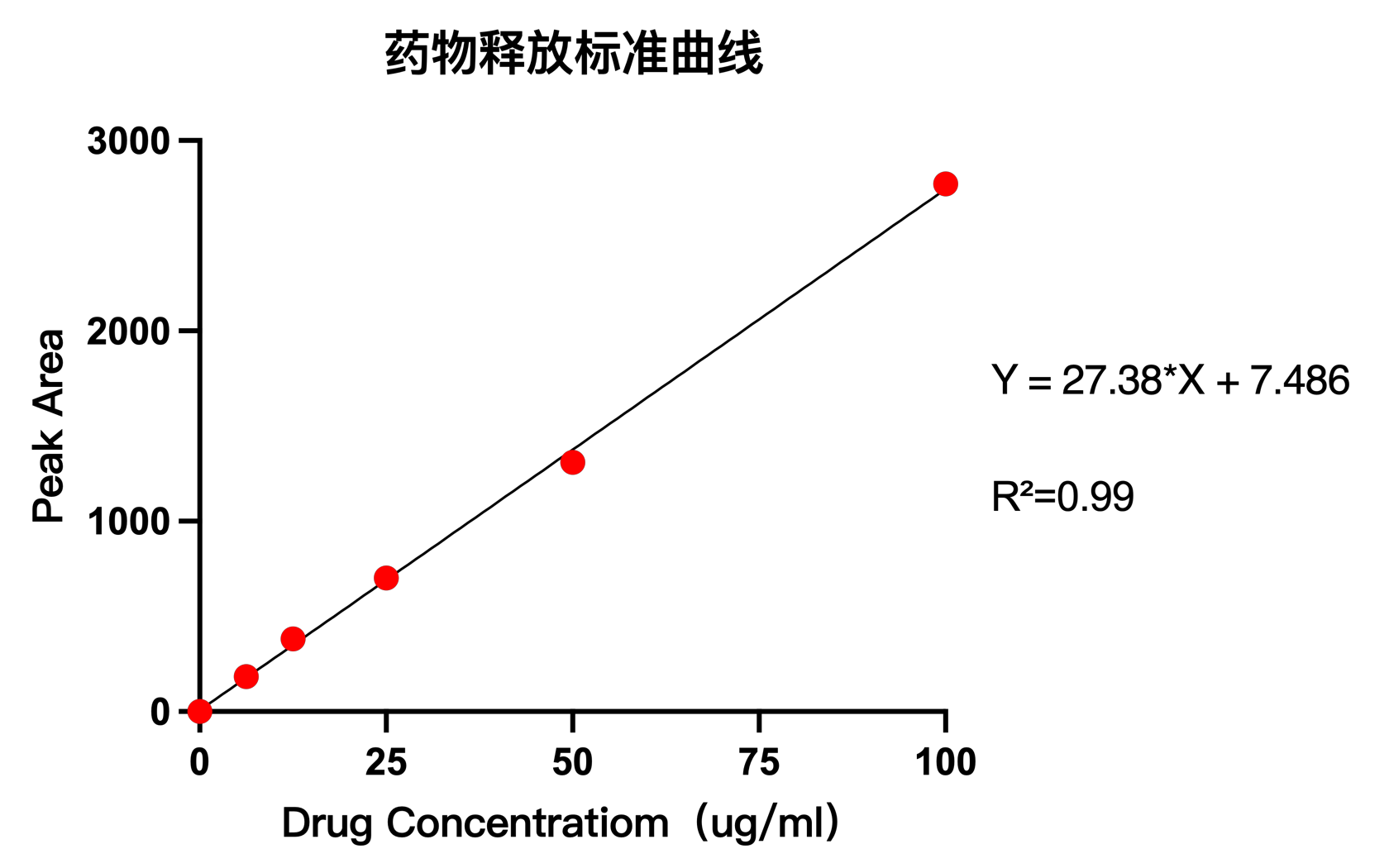


Standard curve for dexamethasone (DEX) in drug release assays

**S2.**


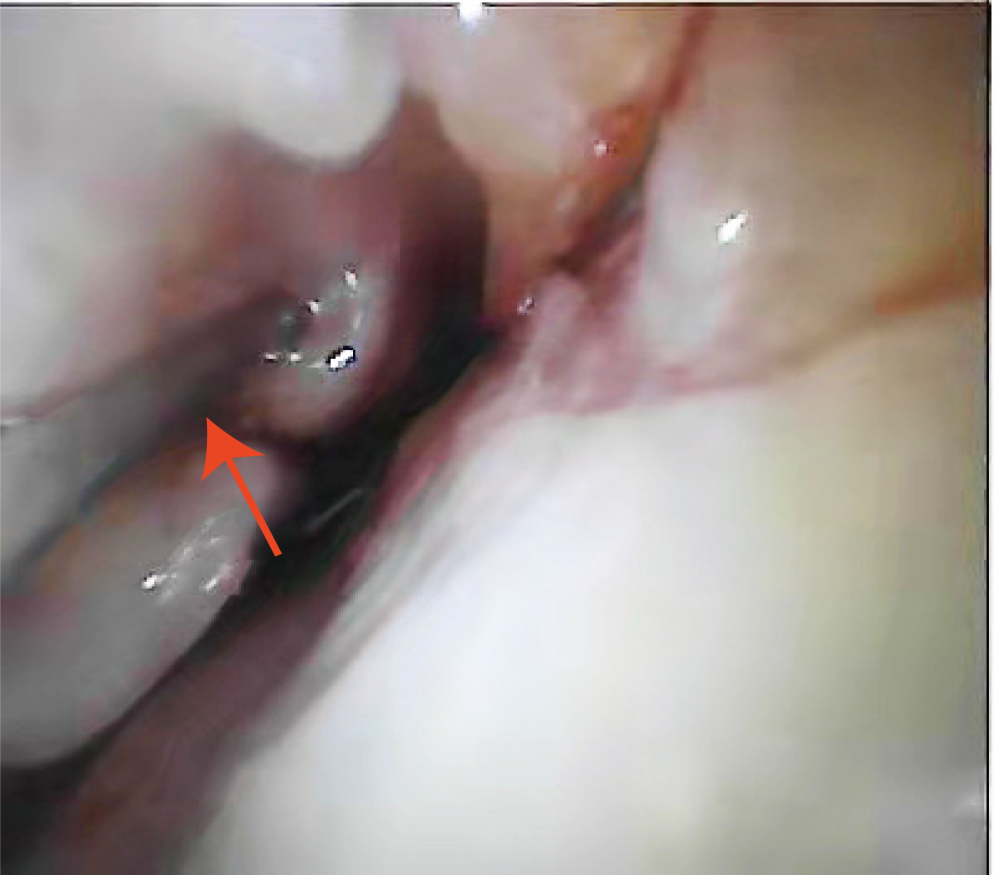


Schematic diagram of endoscopic mucosal injection of DEX-Gel, with the arrow pointing to the injection needle.

**S3.**


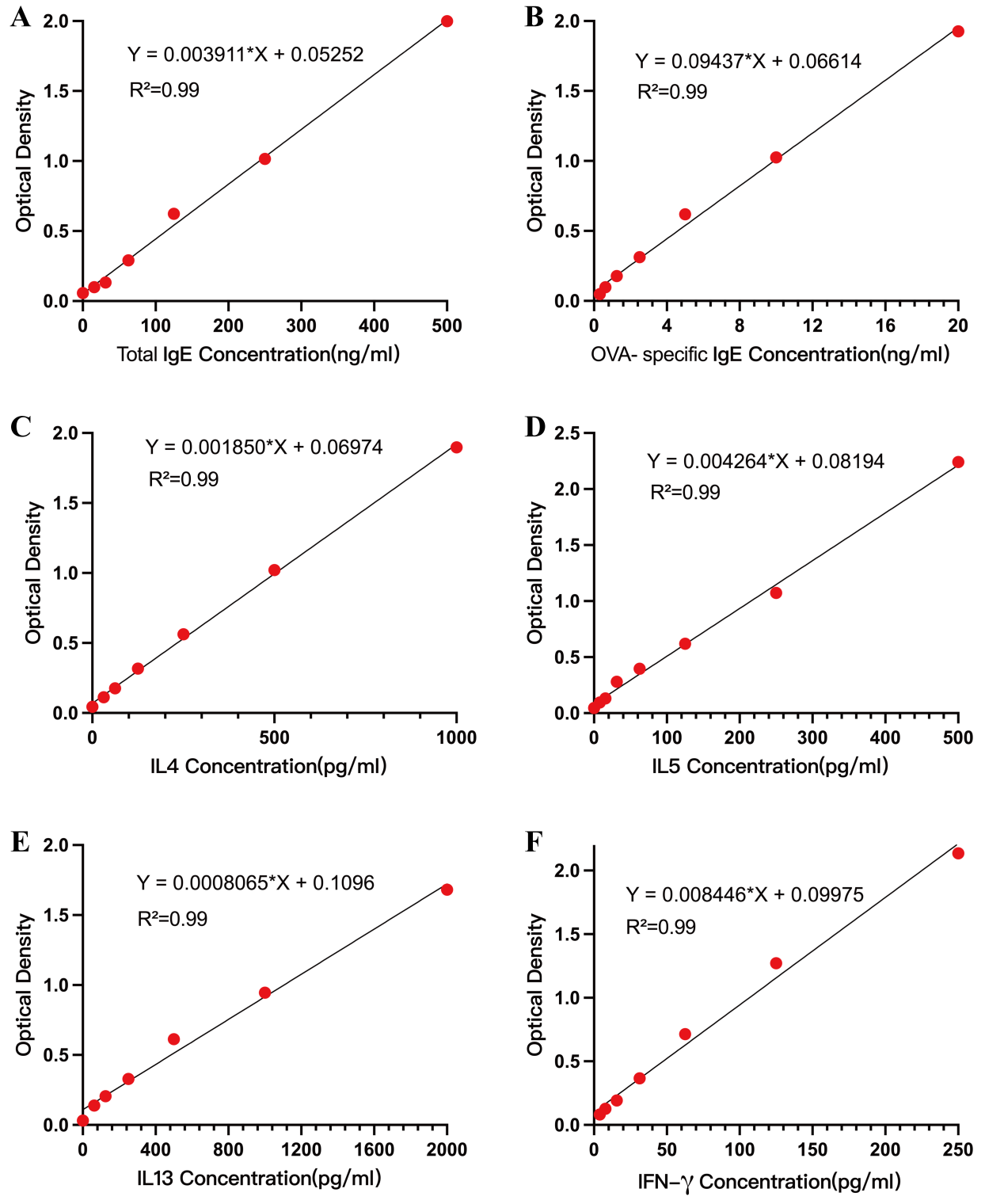


Standard curve of total IgE (A)、OVA-specific IgE (B)、IL4 (C)、IL5 (D)、IL13 (E) and IFN-γ (F) in ELISA assays.

| Gene |  | Sequence |
| --- | --- | --- |
| IL4 | F | CTTCCCTGGCATCTGGAGT  GATGCAGGAGATGCTGACAC |
|  | R |  |
| IL5 | F | TCAGGGGCTAGACATACTGAAG  CCAAGGAACTCTTGCAGGTAAT |
|  | R |  |
| IL13 | F | TGAGCAACATCACACAAGACC  GGCCTTGCGGTTACAGAGG |
|  | R |  |
| Eotaxin | F | GAATCACCAACAACAGATGCAC  ATCCTGGACCCACTTCTTCTT |
|  | R |  |
| IL17 | F | GGCCCTCAGACTACCTCAAC  TCTCGACCCTGAAAGTGAAGG |
|  | R |  |
| IFN-γ | F | GCCACGGCACAGTCATTGA  TGCTGATGGCCTGATTGTCTT |
|  | R |  |
| AQP5 | F | GCCCTCTTAATAGGCAACCAG  GCATTGACGGCCAGGTTAC |
|  | R |  |
| GAPDH | F | TGGCCTTCCGTGTTCCTAC  GAGTTGCTGTTGAAGTCGCA |
|  | R |  |

**S4.**

Primer sequences for different genes. Gene expression levels were measured by the 2-ΔΔCT method.

.

**S5.**

|  | P1 | P2 | P3 | P4 | P5 | P6 | P7 | P8 | P9 |
| --- | --- | --- | --- | --- | --- | --- | --- | --- | --- |
| F127(mg/ml) | 10 | 10 | 10 | 20 | 20 | 20 | 30 | 30 | 30 |
| SA (mg/ml) | 10 | 20 | 30 | 10 | 20 | 30 | 10 | 20 | 30 |
| Maxium Dexamethasone load(mg) | 16 | 18 | 18 | 20 | 24 | 26 | 28 | 30 | 30 |

Two-factor, three-level orthogonal experiment, P9 prescription maximum loadable dexamethasone 30 mg.

S6


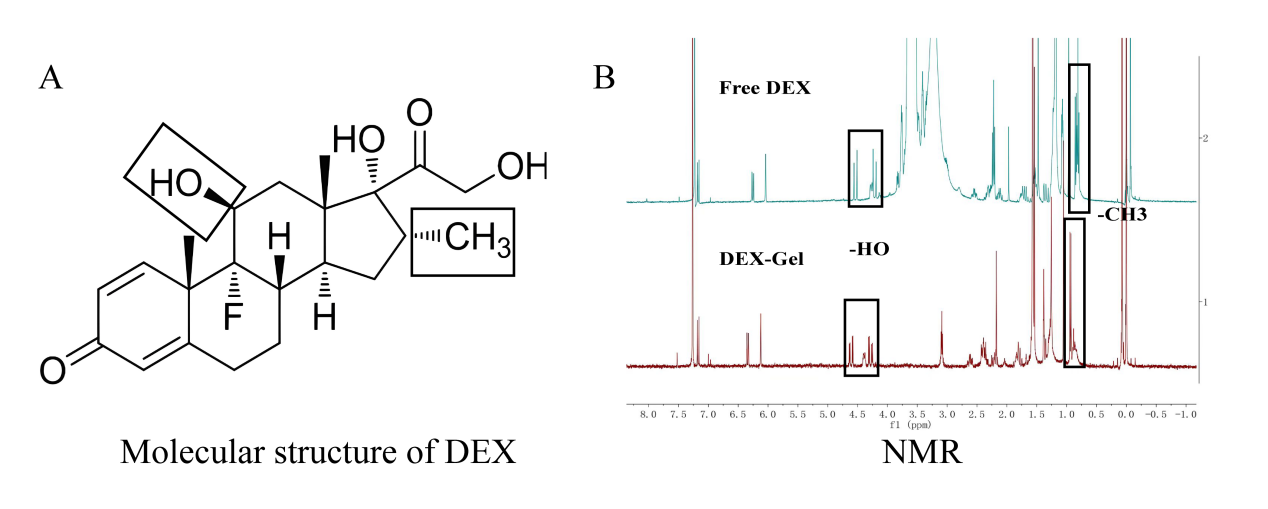


Molecular structure of DEX，with the active groups shown in the black rectangle(A) .NMR detection of Free DEX and DEX-Gel, with the active groups shown in the black rectangle(B).

**S7.**

**
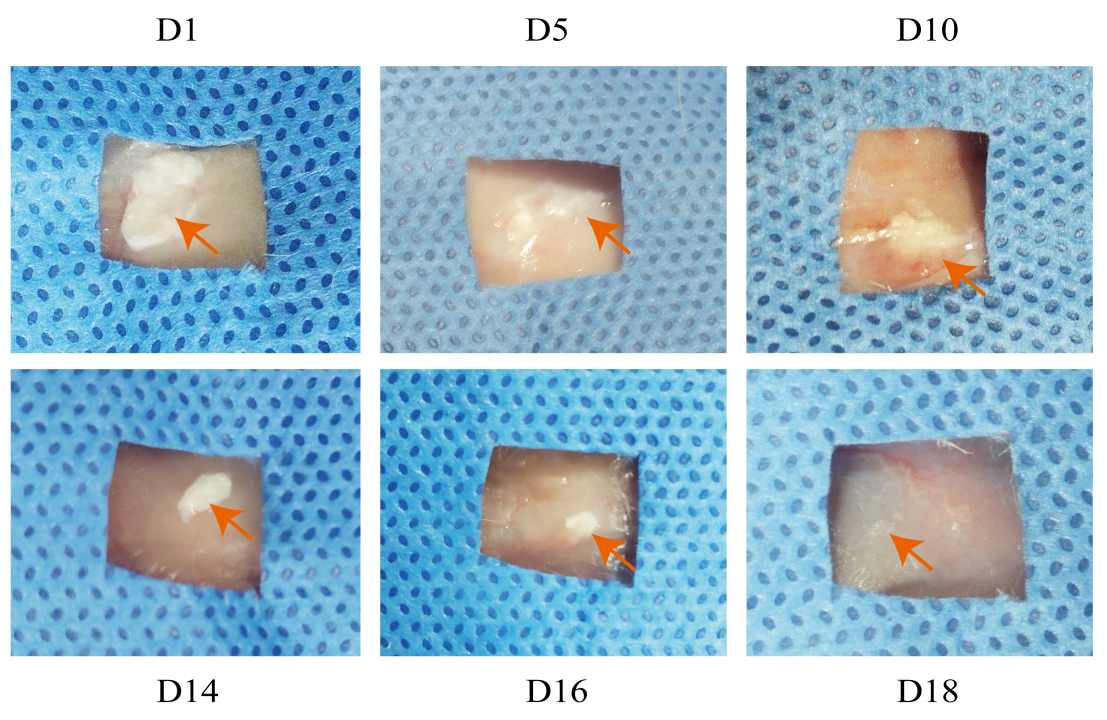
**

DEX-Gel in vitro degradation experiments, where yellow arrows show DEX-Gel

**S8.**


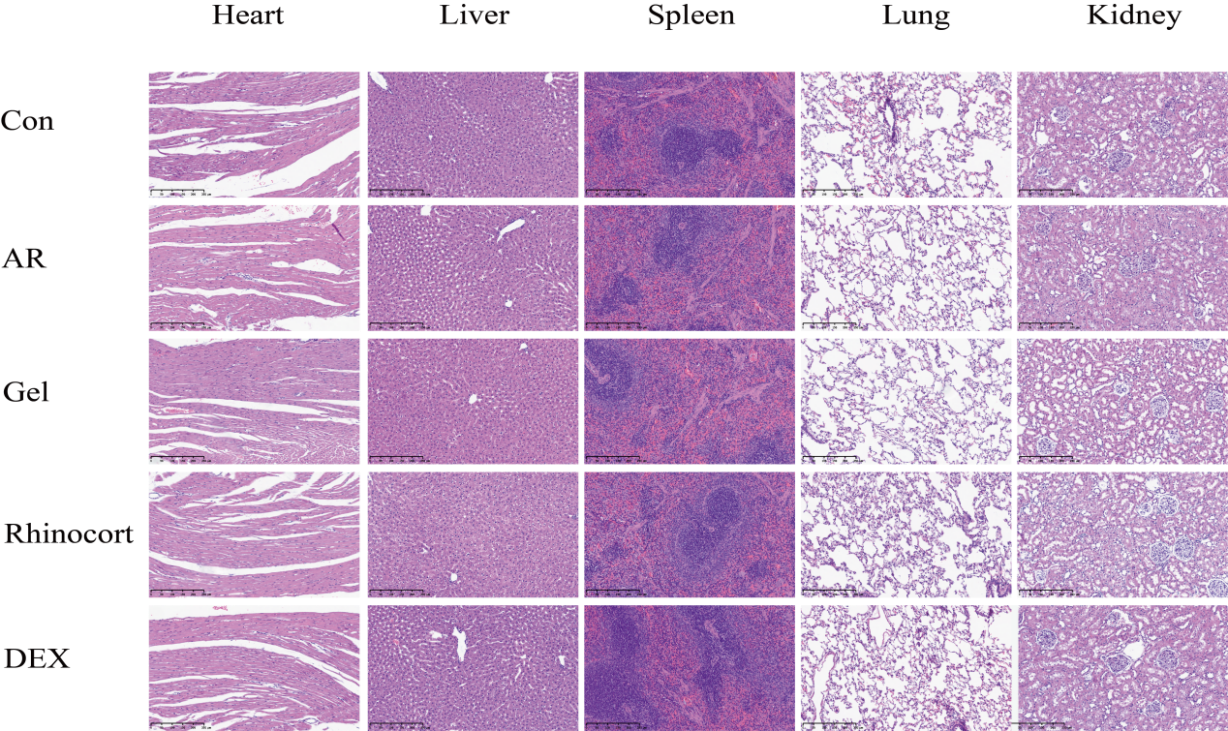


HE staining of the main organs of SD rats in the groups Con、AR、Gel、Rhinocort and DEX. Scare bars = 250 µm.

**S9.**

| Score | 0 | 1 | 2 | 3 |
| --- | --- | --- | --- | --- |
| Nasal rubbing | < 2times | 2 to 4 times | 5 to 10 times | >10times |
| Sneezing | < 2times | 2 to 4 times | 5 to 10 times | >10times |
| Nasal flow | none | one nostril | both nostrils | out-flowing |

Allergy score in rat model of AR, total score >5 considered successful modeling.

**S10.**


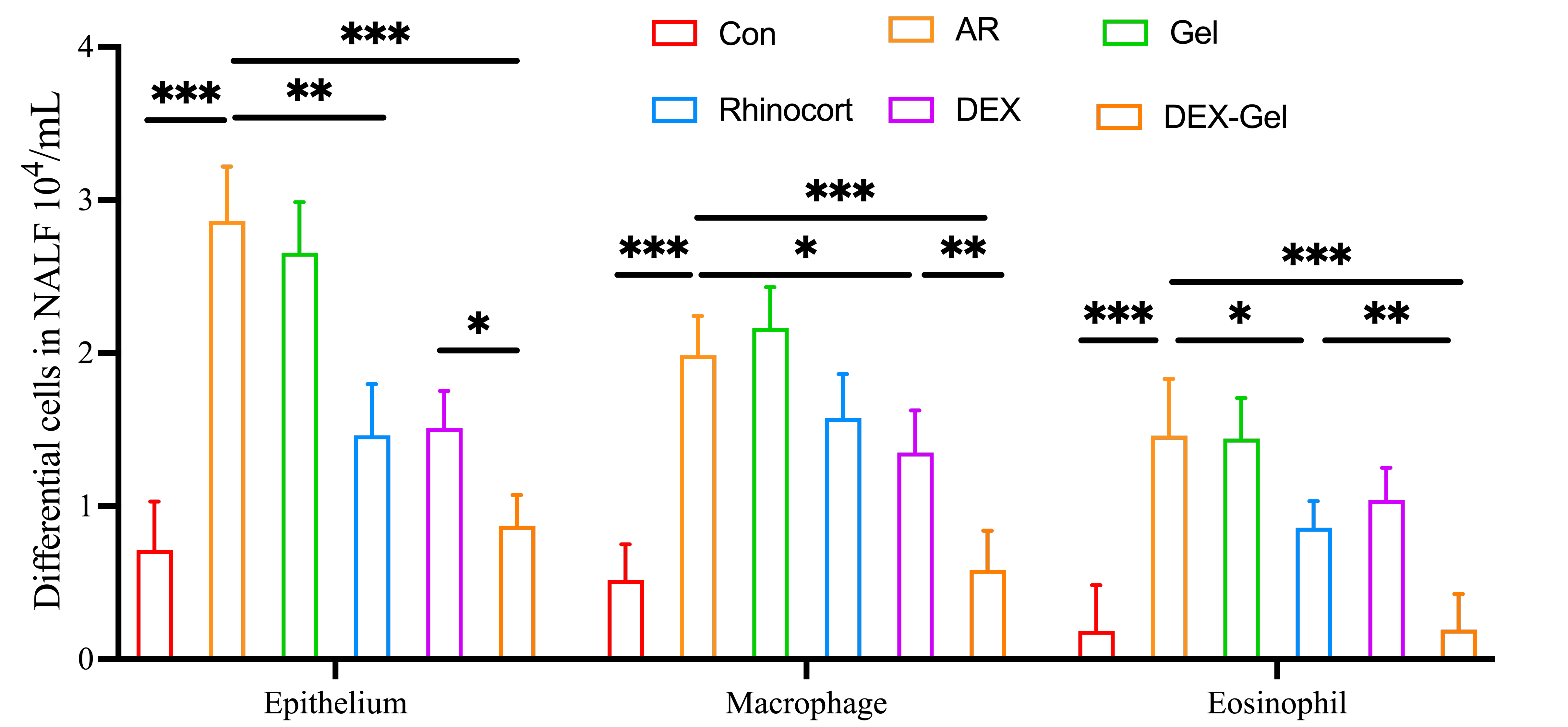


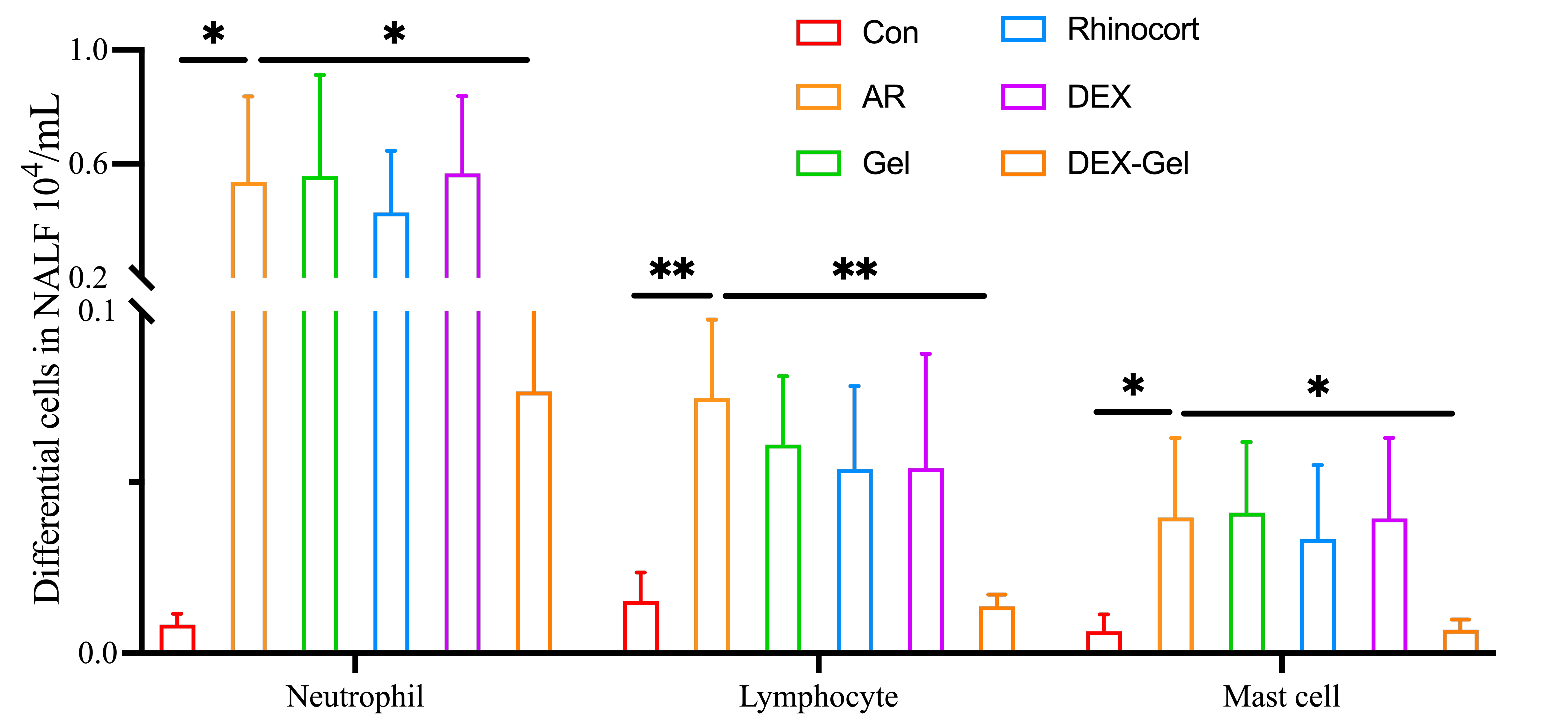


Number of different cells in nasal lavage fluid. Data are expressed as mean ± SD. *P < 0.05, **P < 0.01 and ***P < 0.001, significantly different from the ANOVA group.

**S11.**


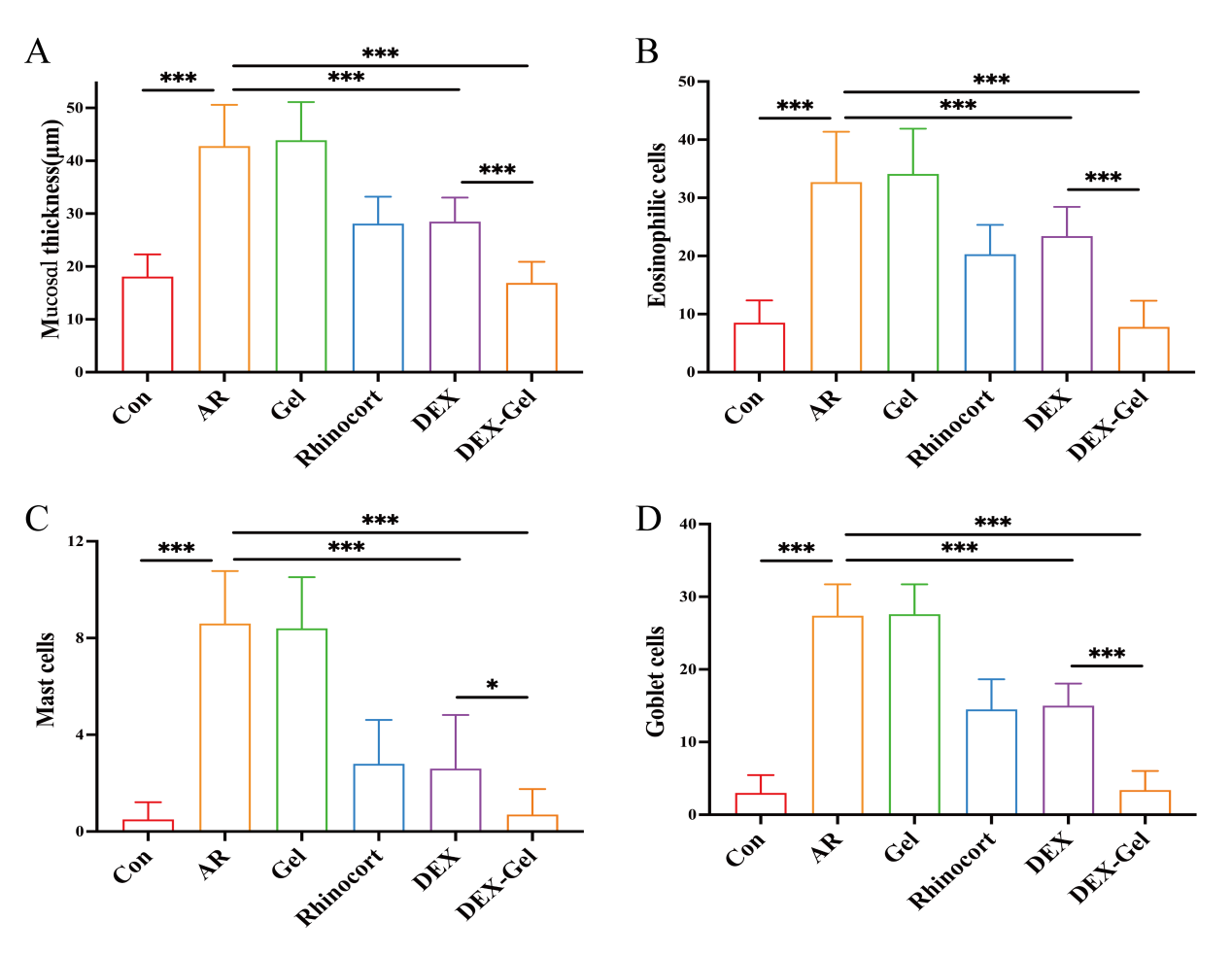


Mucosal thickness (A), number of eosinophils (B), mast cells (C) and goblet cells (D) in groups Con, AR, Gel, Rhinocort, DEX and DEX-Gel. Data are expressed as mean ± SD. *P < 0.05, **P < 0.01 and ***P < 0.001, significantly different from the ANOVA group.

**S12.**


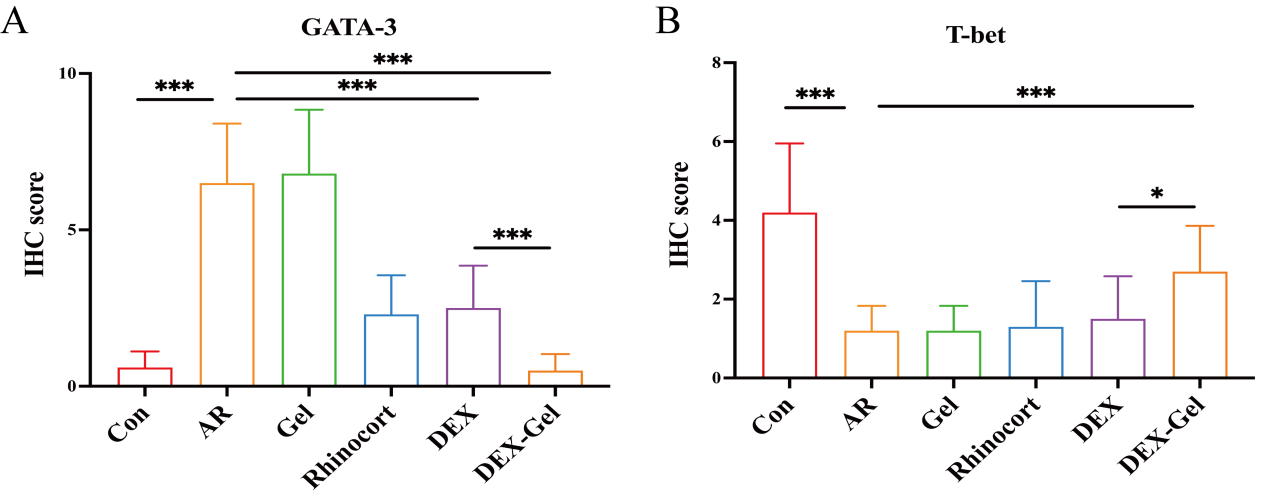


Immunohistochemical scores for GATA-3 (A) and T-bet (B) in groups Con, AR, Gel, Rhinocort, DEX and DEX-Gel. The scoring system included assigning scores based on the percentage of positive cells: 0 (<5%), 1 (5-25%), 2 (25-50%), 3 (50-75%), and 4 (>75%). Staining intensity was also scored: 0 (colorless), 1 (light yellow), 2 (tan), and 3 (brown). The total score was determined by multiplying the positive cell score with the staining intensity score. Data are expressed as mean ± SD. *P < 0.05, **P < 0.01 and ***P < 0.001, significantly different from the ANOVA group.
